# Genome-wide characterization of cys-tathionine-β-synthase domain-containing proteins in sugarcane reveals their role in defense responses under multiple stressors

**DOI:** 10.3389/fpls.2022.985653

**Published:** 2022-08-25

**Authors:** Jing-Ru Zhou, Juan Li, Jia-Xin Lin, Hui-Mei Xu, Na Chu, Qin-Nan Wang, San-Ji Gao

**Affiliations:** ^1^National Engineering Research Center for Sugarcane, Fujian Agriculture and Forestry University, Fuzhou, China; ^2^Institute of Nanfan and Seed Industry, Guangdong Academy of Sciences, Guangzhou, China

**Keywords:** CBS domain containing proteins (CDCPs), sugarcane, gene expression, *Acidovorax avenue* subsp. *avenae*, abiotic stress, defense response

## Abstract

Cys-tathionine-β-synthase (CBS) domain-containing proteins (CDCPs) are essential for regulating plant responses to various biotic and abiotic stressors. This study describes the systematic identification and characterization of CDCP family genes in *Saccharum spontaneum*. A total of 95 *SsCDCP* genes and eight phylogenetic groups were identified that were distributed over 29 chromosomes of the AP85-441 genome. Most (78/95) *SsCDCPs* underwent fragment duplication events, and 64 gene pairs were located in synteny blocks. Expression profiling of nine *ShCDCP*s was also carried out in the *Saccharum* spp. cultivars ROC22 and MT11-611 that are resistant and susceptible to red stripe, respectively, in response to: (i) Infection by the bacterial pathogen *Acidovorax avenue* subsp. *avenae* (*Aaa*); (ii) abiotic stressors (drought and salinity); and (iii) exogenous salicylic acid (SA) treatment. Members of one gene pair (*ShCBSD-PB1-5A* and *ShCBSD-PB1-7A-1*) with a fragment duplication event acted as negative regulators in sugarcane under four stresses, as supported by the significantly decreased expression levels of *ShCBSD-PB1-5A* (23–83%) and *ShCBSD-PB1-7A-1* (15–75%) at all-time points, suggesting that they have functional redundancy. Genes in another pair, *ShCBS-4C* and *ShCBS-4D-1*, which have a fragment duplication event, play opposing regulatory roles in sugarcane exposed to multiple stresses, particularly *Aaa* and NaCl treatments. *ShCBS-4C* expression was significantly decreased by 32–77%, but *ShCBS-4D-1* expression was dramatically upregulated by 1.2–6.2-fold in response to *Aaa* treatment of both cultivars across all-time points. This result suggested that both genes exhibited functional divergence. Meanwhile, the expression of *SsCBSDCBS-5A* was significantly upregulated in ROC22 by 1.4–4.6-fold in response to the four stressors. These findings provide important clues for further elucidating the function of *ShCDCP* genes in sugarcane responding to a diverse range of stresses.

## Introduction

Climate change is leading to more frequent extreme environmental events, such as abiotic (drought, low and high temperature, high salinity, and soil salinization) and biotic (invasive arthropod pests and diseases) stressors (González Guzmán et al., 2022). Furthermore, climate change that produces various abiotic stresses has affected the incidence and geographic distribution of plant diseases and pathogens (Burdon and Zhan, 2020). These environmental stressors have major impacts on food production worldwide (González Guzmán et al., 2022; Karthika and Govintharaj, 2022).

Sugarcane (*Saccharum* spp.) is an important global sugar and biofuels crop that is distributed in tropical and sub-tropical areas, where it is subject to various biotic and abiotic stressors (Javed et al., 2020). Among biotic stressors, the bacterial pathogen *Acidovorax avenae* subsp. *avenae* (*Aaa*) that causes red stripe in sugarcane can lead to serious yield reduction and even plant death (Li et al., 2018). Red stripe disease commonly occurs in main sugarcane-planting areas in China, with varying incidences ranging from 4 to 23% in cultivar FN38 (Fu et al., 2017) and 8–80% in cultivar YZ03-194 (Shan et al., 2017). In Argentina, red stripe affected 30% of the milling stems causing serious economic losses (Fontana et al., 2019). In general, plants respond to pathogen infection via two layers of the immune system, pattern-triggered immunity (PTI) and effector-triggered immunity (ETI) (Zhai et al., 2022). Our previous studies using transcriptome (Chu et al., 2020), proteomic (Zhou et al., 2021), and genome-wide analyses (Chu et al., 2022) revealed that multiple genes/proteins and their related metabolites and signal pathways are involved in defense responses in sugarcane.

Abiotic stresses such as drought, salinity, extreme temperature, and low soil fertility also affect sugarcane growth and yield worldwide (Lakshmanan and Robinson, 2014; Budeguer et al., 2021). Drought is the most important abiotic factor and can reduce sugarcane yield by up to 50–60% (Ferreira et al., 2017; Flack-Prain et al., 2021). Generally, plants or organs responding to water stress exhibit physiological and metabolic changes to minimize water loss under moderate-to-severe short-term stress (Lakshmanan and Robinson, 2014; Ferreira et al., 2017). Salinity is another important abiotic factor that negatively affects sugarcane production in many areas (Budeguer et al., 2021). Sugarcane is more sensitive to soil salinity during germination and early growth stages compared to later stages of plant growth (Lakshmanan and Robinson, 2014). Sugarcane plants that are often reported to be moderately salt-sensitive and grow in saline soil have adverse physiological and developmental disruptions (Lakshmanan and Robinson, 2014; Brindha et al., 2019). Many phytohormones, plant growth regulators, and signaling molecules participate in abiotic stress responses in sugarcane (Ferreira et al., 2017; Budeguer et al., 2021).

Cys-tathionine-β-synthase (CBS) domain-containing proteins (CDCPs) are an evolutionarily conserved superfamily of proteins that contain varying numbers of CBS domains (Kushwaha et al., 2009; Hao et al., 2016; Tomar et al., 2022). The CBS domain was originally discovered in the archaebacterium *Methanococcus jannaschii* (Bateman, 1997). The CBS domain contains about 60 amino acid residues that form two α-helices and three β-strands and generally exists as tandem repeats, particularly in pairs or quads, in the polypeptide (Anashkin et al., 2017; Tomar et al., 2022). In addition to the CBS domain, CDCP family genes encode other domains such as CNNM (or DUF21), inosine-5′-monophosphate dehydrogenase (IMPDH), Phox and Bem1 (PB1), voltage chloride channel (Voltage CLC) (Kushwaha et al., 2009; Tomar et al., 2022). Phylogenetic groupings of CDCP family genes differ among plant species. There are eight groups in *Arabidopsis thaliana* (Kushwaha et al., 2009) and *Triticum aestivum* (Guo et al., 2020), and nine in *Oryza sativa* (Kushwaha et al., 2009) and *Glycine max* (Hao et al., 2016). Recently, 14 major clades of CDCP family genes were identified in 11 genomes from ten *Oryza* species (Tomar et al., 2022).

The CBS domain was found to be widely associated with several proteins that have distinct functions such as AMP-activated protein kinase (AMPK), IMPDH, and CLC (Jeong et al., 2013; Labesse et al., 2015; Subba et al., 2021). The activity of related enzymes and transporter domains was shown to be regulated by the CBS domain that mediates binding of adenosine-based molecules such as AMP, ATP or ASM (S-adenosylmethionine) (Kemp, 2004; Baykov et al., 2011; Anashkin et al., 2017). The CBS domain is an efficient regulatory element and is integrated into proteins with different functions to enhance or weaken protein activity depending on the binding of different ligands (Anashkin et al., 2017). Additionally, signals are transmitted remotely between different subunits of AMPK through the CBS domain and allosteric regulation resulting from the CBS domain can be integrated into more complex regulatory mechanism as in AMPK (Anashkin et al., 2017).

Previous studies have showed that CDCP family genes participate in regulation of plant growth and development, environmental stress, and pathogen infection. In *Arabidopsis*, some CDCPs in root and shoot tissues are expressed in response to drought, salinity, and wounding stresses (Kushwaha et al., 2009); *AtCBSX1* was found to modulate development by regulating the thioredoxin system in chloroplasts (Yoo et al., 2011), whereas *AtCBSX3* is involved in plant development and the redox system through regulation of the generation of reactive oxygen species (ROS) in mitochondria (Shin et al., 2020). In *Oryza sativa*, *OsBi1* overexpression can enhance plant resistance against the herbivore brown planthopper (*Nilaparvata lugens* Stal.) (Wang et al., 2004). Some genes encoding *CDCPs* from rice were found to be involved in responses to multiple stresses such as drought, salinity, and wounding (Kushwaha et al., 2009). *OsCBSX4* overexpression in tobacco plants confers strong resistance to salinity stress (Singh et al., 2012). *OsCBSX9* and *OsCBSCBS4* displayed significantly higher expression levels under both salinity and drought stress conditions in rice plants (Tomar et al., 2022). For *Glycine max*, *GmCBS21* and *GmCBSDUF3* overexpression in *Arabidopsis* showed that the transgenic plants had enhanced tolerance to low nitrogen stress (Hao et al., 2016) and to drought and salt stress (Hao et al., 2021). A recent iTRAQ proteomic analysis revealed that expression of a CBS domain-containing protein was upregulated in wheat (*T. aestivum*) in response to waterlogging (Yang et al., 2022).

Modern sugarcane cultivars with an allo-autopolyploid genome contain chromosomes from *S. officinarum* (80%) and *S. spontaneum* (10%) (D’Hont et al., 1996), but the characteristics of stress response, disease resistance, and regeneration ability of these cultivars are derived from *S. spontaneum* (Garsmeur et al., 2018). To better understand features of the sugarcane genome, two reference genome sequences from the *S. spontaneum* clones AP85-441 (Zhang et al., 2018) and Np-X (Zhang et al., 2022) were assembled at the chromosome level, while three draft genome sequences from hybrid genotypes R570 (Garsmeur et al., 2018), SP80-3280 (Souza et al., 2019), and CC01-1940 (Trujillo-Montenegro et al., 2021) were assembled at a non-chromosome level. These sugarcane genome sequences, particularly in AP85-441 and Np-X, are convenient for exploring how resistance genes are related to various stress responses in sugarcane. It may be hypothesized that some members of the CDCP gene family are involved in alleviating biotic and abiotic stresses in sugarcane. However, systematic identification and analysis of the CDCP gene family in sugarcane remains incomplete. Thus, the objectives of this study were: (i) Identification and characterization of CDCP family genes in *S. spontaneum* AP85-441; (ii) investigation of expression profiles in ROC22 and MT11-610 cultivars after *Aaa* inoculation, and drought, salinity as well as exogenous salicylic acid (SA) treatment; and (iii) comparison of functional redundance and divergence of CDCP family genes in stress responses. Our results provide important information about several CDCP genes and how they are involved in response to different stressors.

## Materials and methods

### Plant growth and experimental treatments

The sugarcane cultivars ROC22 (resistant to red stripe) and MT11-610 (susceptible to red stripe) were provided by the National Engineering Research Center for Sugarcane, Fujian Agriculture and Forestry University, Fuzhou, China (26.0849°N, 119.2397°E). Single buds cut from the two varieties were immersed in flowing water (24 h) and then dipped in hot water at 50°C for 2 h. Sugarcane plants were maintained for 28 days in a growth chamber at 28°C and 60% relative humidity under a 16/8 h photoperiodic cycle. Four stress experiments were performed following the procedure described by Chu et al. (2022). Briefly, the *Aaa* strain SC-026 (10^8^ CFU/ml) was used for sugarcane seedling inoculation with a leaf-cutting method (Chu et al., 2020). The plants were inoculated with liquid NB medium as a control (CK). Leaf samples were collected at 0, 24, 48, and 72 h post-inoculation (hpi) to examine responses to *Aaa* stress. Sampling points for seedlings treated with 25% PEG6000 were 0, 3, 6, and 12 h; sampling points for 250 mM NaCl or 0.1 mmol/L SA (containing 0.01% Tween-20) treatments were 0, 6, 12, and 24 h after treatment. Three biologic replicates of six plants were used at each sampling time.

### Identification of cys-tathionine-β-synthase domain-containing proteins in *Saccharum spontaneum*

*A. thaliana* and *T. aestivum* CDCP protein sequences were obtained from TAIR^1^ and UniProt,^2^ respectively. These sequences were used as bait to search for genes encoding CDCP proteins in the AP85-441 *S. spontaneum* genome^3^ using NCBI BLAST-P^4^ with an *e*-value < 1e^–5^ (default parameters were used for other settings). Sequences remaining after manual removal of redundant sequences were used for further analysis. PF00571 (CBS domain) and HMMER software with default parameters were used to search for candidate CDCP genes in *S. spontaneum*. Furthermore, the Conserved Domain Search,^5^ Pfam online tools^6^ and SMART online tools^7^ with default settings were used to verify each gene containing a CBS domain. The CDCP family genes identified in *S. spontaneum* were termed *SsCDCPs*. The nomenclature of all CDCP family genes identified in *S. spontaneum* and two sugarcane cultivars was identical to *Arabidopsis* (Kushwaha et al., 2009) and *Triticum aestivum* (Guo et al., 2020). For example, in the *SsCBS-3C* gene “Ss” is an abbreviation for *S. spontaneum*, “CBS” stands for the typical conserved domain, and “3C” refers to the chromosomal location.

### Analysis of physico-chemical properties

The ExPASy Proteome Server^8^ was used to compute physio-chemical traits such as the number of amino acids (aa), molecular weight (MW), and theoretical isoelectric points (pI). The Plant-mPLoc^9^ online tool was used to predict the sub-cellular localization of SsCDCP members.

### Multiple sequence alignment and phylogenetic analysis

Alignment of 256 CDCP protein sequences (*A. thaliana* = 34, *T. aestivum* = 127, and *S. spontaneum* = 95) was performed using the CLUSTALW program implemented in the MEGA version 11 (Tamura et al., 2021). A phylogenetic tree was constructed using MEGA version 11 with the maximum likelihood (ML) method and bootstraps of 1,000 replicates. Visualizations were generated using the EvoIView server.^10^

### Gene structure, *cis*-regulatory elements, and gene duplication analysis

Conserved motifs of SsCDCP sequences were analyzed with the MEME tool^11^ with default parameters. Conserved domains were checked with NCBI-CDD (Use default parameters) (see text footnote 5) (Lu et al., 2020). The gene structure of *SsCDCPs* was visualized using TBtools v0.6655 (Chen et al., 2020). *Cis*-acting elements in the promoter sequences (2,000 bp) of each *SsCDCP* gene were analyzed using PlantCARE online software^12^ with default parameters. TBtools v0.6655 was used to analyze and visualize the gene duplication events among *SsCDCP* genes (Wang et al., 2012).

### Expression profiling using ribonucleic acid-seq data

To determine the expression profiling of CDCP genes in sugarcane cultivars following *Aaa* infection, a previously published ribonucleic acid (RNA)-seq dataset (PRJNA579959) was used (Chu et al., 2020). The *CDCP* genes identified in the *Saccharum* spp. hybrid were termed *ShCDCPs*. The expression abundance of the *ShCDCP*s was calculated using the fragments per kilobase of transcript per million fragments mapped (FPKM) value and the relative expression level is shown as log_2_ (Fold Change) values.

### *ShCDCP* gene expression analysis by RT-qPCR

Transcript expression of nine *ShCDCP* genes was investigated by RT-qPCR assay. Total RNA was extracted from leaf samples and cDNA was synthesized by reverse transcription as previously described (Chu et al., 2020). Primers for nine *ShCDCP* genes were designed with Primer 5.0 software (Supplementary Table 1). The SYBR green dye method was used for RT-qPCR amplification (Javed et al., 2022). Glyceraldehyde 3-phosphate dehydrogenase (*GAPDH*) was used as a reference gene. The 2^–ΔΔCt^ quantification method was used to determine the expression of each *ShCDCP* gene. Each sample was assayed with three biological and three technical replicates.

### Statistical analysis

The relative expression levels at each time point were analyzed by one-way ANOVA and the test of significance among means was carried out with LSD (least significance difference) at 5% probability level (*p* ≤ 0.05). All statistical analyses were carried out using SPSS software (IBM SPSS Statistics 25).

## Results

### Identification and physico-chemical properties of cys-tathionine-β-synthase domain containing proteins family genes in *Saccharum spontaneum*

A total of 37 *SsCDCP* genes with varying numbers of alleles were identified in *S. spontaneum* clone AP85-441. Among these *SsCDCPs*, 21.6% (8/37) lacked alleles, and the remainder had 1–6 alleles (Supplementary Table 2). Thus, a total of 95 *SsCDCP* genes were identified in AP85-441. The physico-chemical properties showed that the 95 SsCDCP proteins had: between 169 (SsCBS-3A-1) and 1,367 (SsCBS-SIS-4C) amino acids; molecular weights ranging from 17,138.29 (SsCBS-3A-1) to 147,232.08 (SsCBS-SIS-4C) Da; and isoelectric points between 4.54 (SsTlyc-1C-1) and 10.68 (SsCBS-5A) (Supplementary Table 3). Most (37.9%) *SsCDCPs* were predicted to localize to the plasma membrane, and the remainder were predicted to localize to the chloroplast (30.5%), cytoplasm (16.8%), and mitochondria (10.5%). Only a few *SsCDCPs* (4.2%) localized to the nucleus.

### Phylogeny of cys-tathionine-β-synthase domain containing proteins family genes in three plant species

**FIGURE 1:**
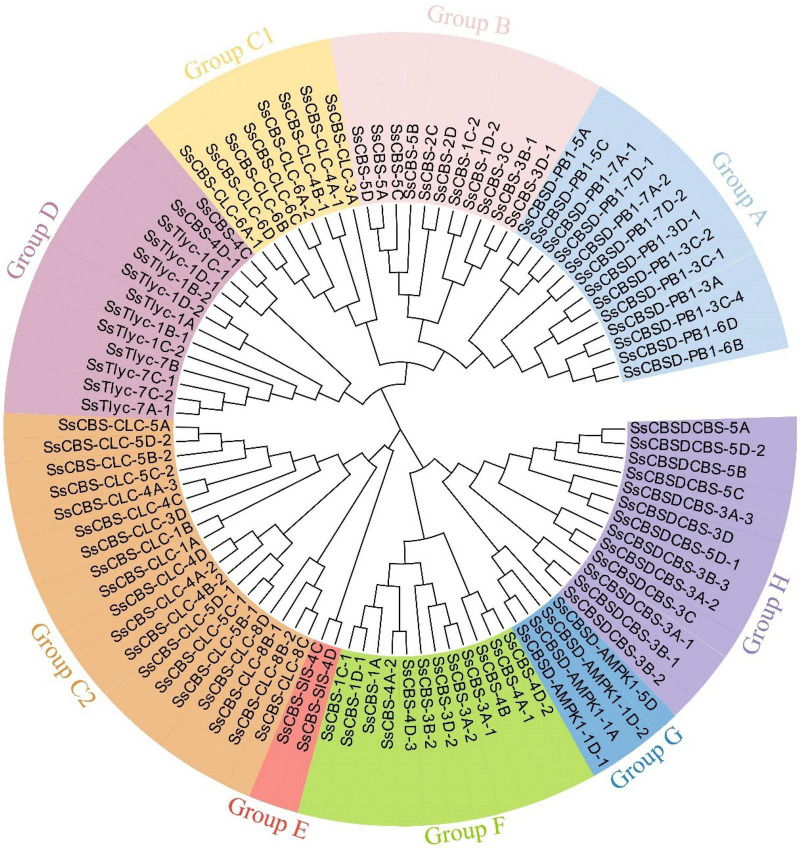
Unrooted phylogenetic tree of *SsCDCPs* identified in *S. spontaneum* AP85-441. The tree was conducted using the maximum likelihood (ML) method with 1,000 bootstrap replicates.

A phylogenetic analysis to describe evolutionary relationships among *S. spontaneum (95 SsCDCPs)*, *A. thaliana* (34 *AtCDCPs*), and *T. aestivum* (127 *TaCDCPs*) showed that all 256 CDCPs could be classified into nine different groups (Supplementary Figure 1). Most (69) CDCPs were in group C, followed by group A (48), and group J had the fewest CDCPs (3). Another phylogenetic tree revealed that all 95 *SsCDCPs* could be divided into eight groups (lack of group J) as compared to all CDCPs identified in the three species. Notably, group C contained 27 CDCPs and could be sub-divided into two groups (C1 and C2) having 8 and 19 genes, respectively (Figure 1).

### Gene structure analysis among *SsCDCP* genes

**FIGURE 2:**
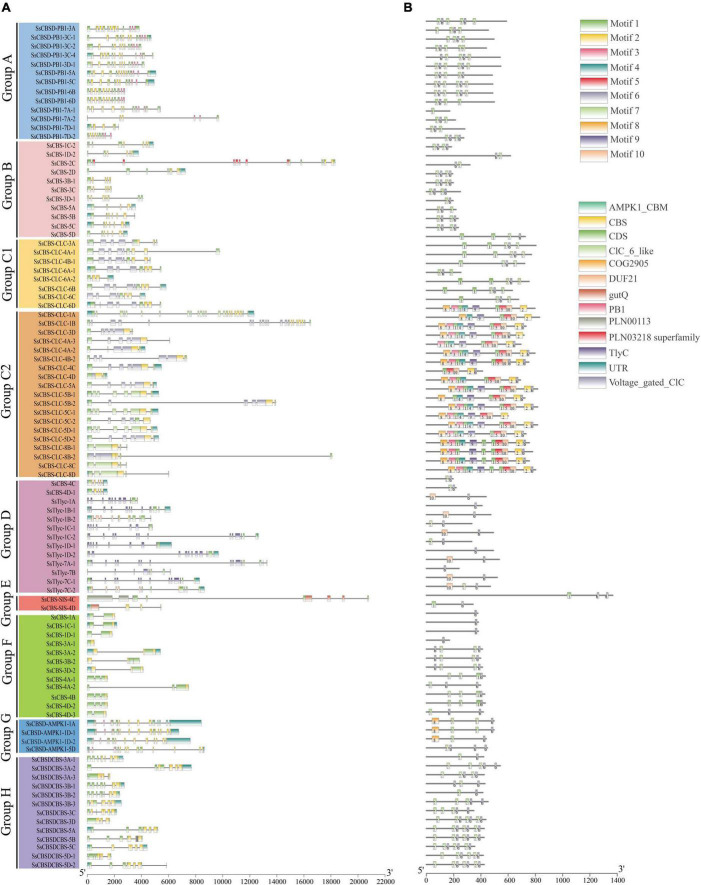
Gene structure **(A)** and conserved motif **(B)** of *SsCDCPs* in *S. spontaneum*. **(A)** Untranslated 5′- and 3′-regions, CDS and conserved domains are displayed with different colored boxes. **(B)** Motifs 1–10 are displayed as different colored boxes. Gene and protein lengths can be estimated using the scales at the bottom.

Among 95 *SsCDCPs*, 18 had only 5′-UTR, 21 had only 3′-UTR, and 21 *SsCDCPs* had both 5′-UTR and 3′-UTR. The other remaining genes had no 5′-UTR or 3′-UTR (Figure 2 and Supplementary Table 3). The number of introns ranged from 0 (*SsCBS-3A-1*) to 22 (*SsCBS-CLC-1A*), while the number of exons ranged from 1 (*SsCBS-3A-1*) to 23 (*SsCBS-CLC-1A*). *SsCBS-CLC-8B-2* had the longest intron, followed by *SsCBS-SIS-4C* and *SsCBS-CLC-5B-2*. The longest exon structure was observed in *SsCBSD-AMPK1-1A*, followed by *SsCBS-SIS-4C* and *SsCBSD-AMPK1-1D-2*. Among eight *SsCDCP* groups, members of group B (11 *SsCDCPs*), group F (12 *SsCDCPs*) and group H (13 *SsCDCPs*) contained 1–3 CBS domains. The members of other groups had additional domains other than the CBS domain. For example, all group A (13 *SsCDCPs*) members, except for *SsCBSD-PB1-7D-*1, had an additional Phox/Bem1 (PB1) domain. Genes in groups C1 (8 *SsCDCPs*) and C2 (19 *SsCDCPs*) had an additional CLC domain (except for *SsCBS-CLC-6A-2*). Those in Group D (13 *SsCDCPs*) had an additional Tlyc domain (except for *SsCBS-1D-2* and *SsCBS-5D*). *SsCBS-4C* clustered in group D had a unique COG2905 domain. Group E (2 *SsCDCPs*) contained an additional gutQ domain. Group G (4 *SsCDCPs*) had an additional AMPK1_CBM domain (Figure 2A). Conserved motif numbers ranged from 1 (*SsCBSD-PB1-7A-2*) to 10 (*SsCBS-CLC-4B-2*). Motifs 6 and 7 were found in 96 and 65% of *SsCDCPs*, respectively (Figure 2B).

### *In silico* promoter analysis of *SsCDCP* genes

**FIGURE 3:**
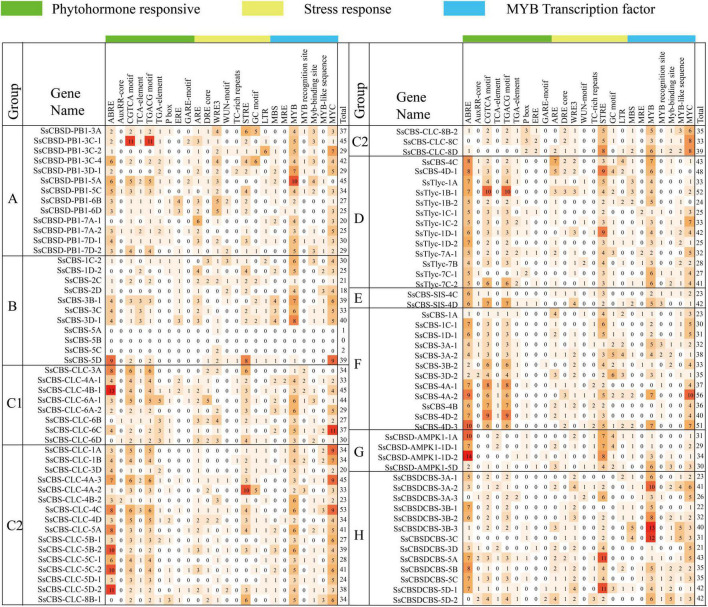
Heatmap of *cis*-regulatory elements in promoter sequences (2 kb) of *SsCDCPs* from *S. spontaneum*.

A total of 24 *cis*-acting regulatory elements related to phytohormones, stress and MYB transcription factors were predicted (Figure 3). Potential functions of *cis*-acting regulatory elements are annotated in Supplementary Table 4. Promoter sequences (2 kb) of *SsCDCPs* contained different numbers of *cis*-elements, ranging from 0 (*SsCBS-5B*) to 56 (*SsCBS-4A-2*). The promoters of the two genes *SsCBS-5A/5C* had only 1–2 *cis*-elements. Most (94%) *SsCDCP* gene*s* had MYB, and ABRE, STRE, CGTCA, and TGACG motifs were present in 85–88% of *SsCDCP* genes.

### Chromosomal distribution and gene duplication analysis of *SsCDCP* genes

**FIGURE 4:**
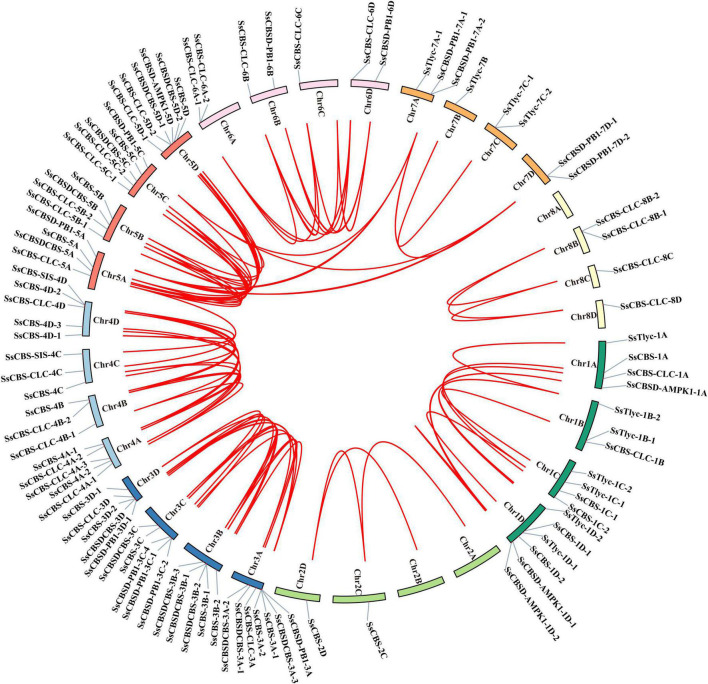
Chromosomal localization and gene replication relationship of *SsCDCPs* in *S. spontaneum*. Ribbon links (Red lines) indicate segmental duplication events between genes. Chromosome numbers are indicated inside the outer circle. The gene names on each chromosome are indicated on the outer circle.

All 95 *SsCDCPs* including 87 alleles were distributed on 29 chromosomes at different densities (Figure 4). Chromosome 3A (Chr3A) had the most *SsCDCPs* (7), followed by Chr1D and Chr5D, which contained six *SsCDCPs*, and then chromosomes 3B, 3C, 3D, 4A, 4D, and 5C that each had five *SsCDCPs*. The other 20 chromosomes had 1–4 *SsCDCPs*. Most *SsCDCPs* were located at the proximal end of each chromosome. Gene duplication and collinear correlation analysis showed that fragment duplication occurred in 82% (78/95) *SsCDCPs* and 64 gene pairs existed in synteny blocks, such as *SsCBSD-PB1-3A* and *SsCBSD-PB1-3C-4*, *SsCBS-4C* and *SsCBS-4D-1*, and *SsCBSD-PB1-5A* and *SsCBSD-PB1-7A-1*. Notably, gene duplication events mainly occurred on chromosomes Chr3A/3B/3C/3D, Chr4A/4B/4C/4D, and Chr5A/5B/5C/5D. The Ka/Ks ratios of all *SsCDCP* gene pairs were < 1, suggesting that they were under purifying selection (Supplementary Table 5).

### Expression analysis of *ShCDCP* gene responses to *Acidovorax avenue* subsp. *avenae* infection

**FIGURE 5:**
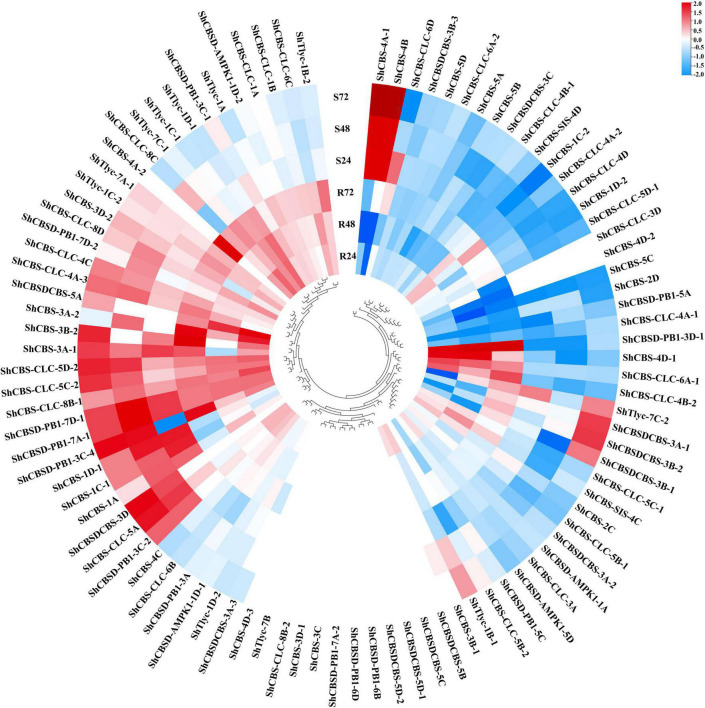
Heatmap of transcript expression profiles of *ShCDCPs* in two cultivars, ROC22 (resistant to red stripe, R) and MT11-610 (susceptible to red stripe, S) based on RNA-Seq data. The gene names are indicated in the outer circle and the expression values mapped to a color gradient from low (blue) to high (red) expression level are shown inside the outer circle.

A total of 83 *ShCDCPs* were identified on the transcriptome database (PRJNA579959). RNA-seq data revealed that these genes showed different expression patterns under *Aaa* infection. Expression levels of 10 *ShCDCPs* were upregulated with an increase of 2% to 2.2-fold, but 16 *ShCDCPs* were downregulated across all-time points in both sugarcane cultivars. Twelve *ShCDCPs* were significantly upregulated in ROC22 (resistant to red stripe) but were downregulated or unchanged in MT11-610 (susceptible to red stripe) across all-time points (Figure 5 and Supplementary Table 6). For example, the transcript level (log_2_FC) of the *ShCBS-4D-1* gene was increased by between 41% and 2.1-fold in ROC22, but this gene was significantly decreased in MT11-610 relative to the control (0 hpi).

**FIGURE 6:**
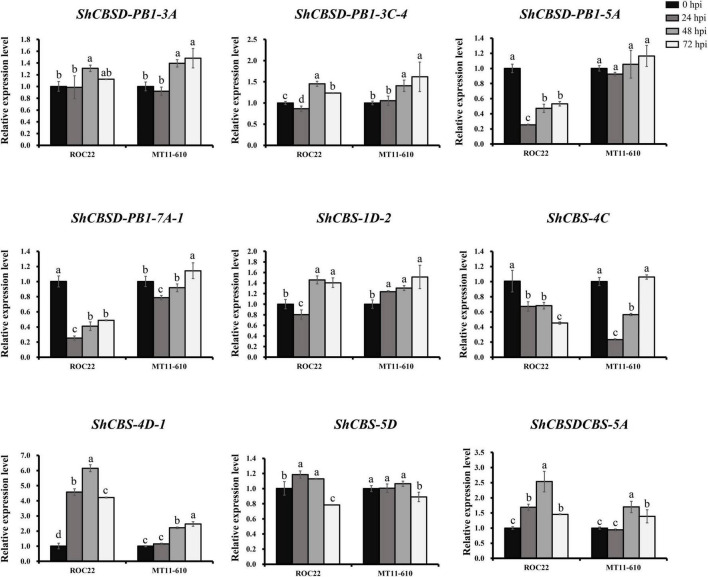
Expression levels of nine *ShCDCPs* in the cultivars ROC22 and MT11-610 with *Aaa* infection based on RT-qPCR data. Means ± standard errors are shown. The same letters atop the bars indicate no significant difference at the 5% level.

Expression patterns of nine *ShCDCPs* were further investigated by RT-qPCR assay in two cultivars after *Aaa* infection (Figure 6 and Supplementary Table 7). In ROC22, expression of *ShCBS-4D-1* and *ShCBSDCBS-5A* was upregulated, while *ShCBSD-PB1-5A*, *ShCBSD-PB1-7A-1*, and *ShCBS-4C* expression was downregulated across all-time points compared to the control (0 hpi). Expression of *ShCBS-4D-1* and *ShCBSDCBS-5A* was increased by more than 4.2-fold and 1.5-fold in ROC22 after *Aaa* infection. In MT11-610, five genes (*ShCBS-1D-2*, *ShCBSD-PB1-3A*, *ShCBSD-PB1-3C-4*, *ShCBS-4D-1*, and *ShCBSDCBS-5A*) were significantly upregulated across all or some timepoints under *Aaa* infection. Three genes (*ShCBSD-PB1-5A*, *ShCBS-4C*, and *ShCBS-5D*) were significantly downregulated or unchanged across all-time points. The *ShCBSD-PB1-7A-1* gene showed a fluctuating expression profile after *Aaa* infection.

### Expression analysis of *ShCDCP* genes under diverse abiotic stresses

**FIGURE 7:**
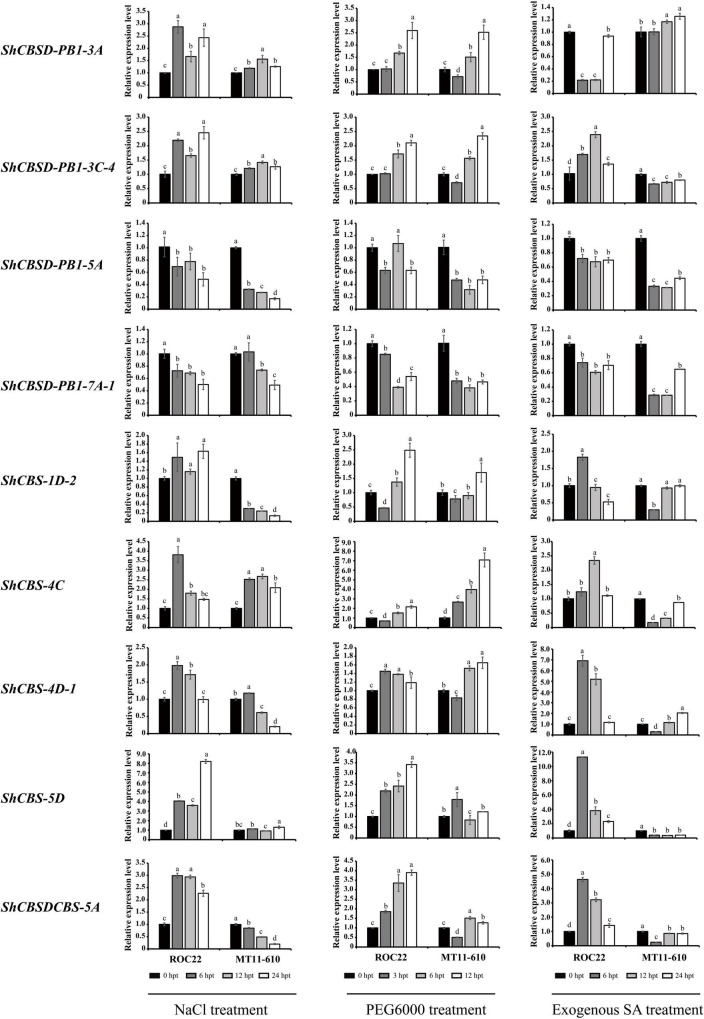
Expression levels of nine *ShCDCPs* in ROC22 and MT11-610 cultivars treated with NaCl, PEG6000 or exogenous SA treatment based on RT-qPCR data. Means ± standard errors are shown. The same letters atop the bars indicate no significant difference at the 5% level. Rows show the same gene and columns correspond to stressor. hpt, h post treatment.

Under NaCl stress, 7/9 *ShCDCPs* shared similar expression profiles in two cultivars, while two genes (*ShCBS-1D-2* and *ShCBSDCBS-5A*) showed cultivar-specific behavior. Three *ShCDCPs* (*ShCBSD-PB1-3A*, *ShCBSD-PB1-3C-4* and *ShCBS-4C*) were significantly upregulated with an increase of 1.2–3.8-fold, while two (*ShCBSD-PB1-5A* and *ShCBSD-PB1-7A-1*) were significantly downregulated in both cultivars across all-time points. *ShCBS-1D-2* and *ShCBSDCBS-5A* were upregulated in ROC22, but downregulated in MT11-610 across all-time points. The expression level of *ShCBS-5D* was increased for the two cultivars except for 12 h post-NaCl treatment in MT11-610. Additionally, in the two cultivars *ShCBS-4D-1* displayed upregulation at early time points (particularly at 6 h) but later was downregulated (Figure 7 and Supplementary Table 7).

Under PEG6000 treatment, two *ShCDCP* genes (*ShCBSD-PB1-5A* and *ShCBSD-PB1-7A-1*) were downregulated in both cultivars across all-time points. However, the other seven genes (except for *ShCBS-5D*) showed downregulation or lower expression levels at early timepoints with PEG6000 treatment (3 h), but thereafter the expression levels progressively increased (Figure 7 and Supplementary Table 7).

Under SA stress, 7/9 *ShCDCPs* shared cultivar-specific expression profiles. Two *ShCDCP* genes (*ShCBSD-PB1-5A* and *ShCBSD-PB1-7A-1*) were downregulated in both cultivars across all-time points, but *ShCBS-1D-2* was also downregulated across all-time points except for 6 h after SA treatment in ROC22. Four genes (*ShCBSD-PB1-3C-4*, *ShCBS-4C*, *ShCBS-5D*, and *ShCBSDCBS-5A*) were dramatically upregulated by 1.1–11.3-fold in ROC22, but were significantly downregulated in MT11-610 at all-time points. The *ShCBSD-PB1-5A* gene was upregulated in ROC22, but downregulated in MT11-610. *ShCBS-4D-1* was upregulated in both cultivars except for 6 h post-SA treatment in MT11-610 (Figure 7 and Supplementary Table 7).

### Functional redundancy and divergence of *ShCDCP* genes responding to various stressors

To investigate the expression profiles of duplicated gene pairs in sugarcane responses to various stressors, three gene pairs with fragment duplication events were examined (Figures 6, 7). Similar transcript profiles and functions existed for the *ShCBSD-PB1-5A* and *ShCBSD-PB1-7A-1* gene pair in the two cultivars under *Aaa* infection and three abiotic stresses, suggesting that the two genes displayed functional redundancy in response to multiple stressors. *ShCBS-4C* and *ShCBS-4D-1* as part of a gene pair displayed different expression patterns in sugarcane under biotic and abiotic stresses, particularly *Aaa* infection, but also with salt and SA treatments, indicating that this gene pair exhibited functional divergence in response to multiple stressors. Two *ShCBSD* genes, *ShCBSD-PB1-3A* and *ShCBSD-PB1-3C-4*, were significantly upregulated in both cultivars under *Aaa*, salt, and drought stresses, but the two genes had opposite roles in individual cultivars following SA treatment.

## Discussion

CDCPs are a large family of proteins that are involved in a variety of biological functions in plants (Hao et al., 2021). Various numbers of CDCP family genes at a genome-wide level were found in previous studies. The number of *SsCDCPs* (95) identified in *S. spontaneum* in this study was lower than that of *T. aestivum* (136) (Guo et al., 2020), but more than that for *A. thaliana* (34), *O. sativa* (59) (Kushwaha et al., 2009), and *Glycine max* (71) (Hao et al., 2016). A large proportion of duplication events (particularly fragment replication) occurred in *SsCDCPs*, which is similar to the *Oryza* species (Tomar et al., 2022) and suggests that duplication events contributed to the expansion of the CDCP gene family in plants. Tandem and fragment duplication events are important driving forces for the evolution of gene families in plants, particular in polyploidy crops (Panchy et al., 2016; Van De Peer et al., 2017).

Various expression patterns of *ShCDCPs* were observed in sugarcane in response to *Aaa* infection revealed by RNA-seq data and RT-qPCR assay. Of these, two genes, *ShCBS-4D-1* and *ShCBSDCBS-5A*, may be associated with resistance to *Aaa* in sugarcane since increased expression levels of both genes were present in ROC22, which is resistant to red stripe, compared to MT11-610, which is susceptible to red stripe. Previous studies showed that proteins containing the CBS domain mostly play a positive regulatory role in resistance to stress and disease in other crops. For example, maximal expression levels of the *TaCDCP1* (*ShCBS-4D-1* homolog) gene were induced in *T. aestivum* inoculated with *Puccinia srtiformis* f. sp. *tritici* at 18 and 96 hpi (Wang et al., 2010). Transcripts of *OsCBSX3* (*ShCBS-4D-1* homolog) were significantly upregulated by inoculation of *M. oryzae* and over-expressing *OsCBSX3* plants exhibited significantly enhanced resistance to *M. oryzae* infection (Mou et al., 2015). Additionally, a *CsCBS* gene could respond positively to cucumber downy mildew and cucumber target leaf spots, as indicated by the continued increase in expression of the *CsCBS* gene in the resistant cultivar D9320 during the early stages and there was no significant change in the susceptible cultivar D0401 (Qin et al., 2018). Our recent studies revealed that the gene encoding the CBS domain containing protein Cluster-13677.239347, which is identical to *ShCBS-4D-1*, plays a role as a positive regulator in sugarcane after infection by *Aaa* based on transcriptome and proteome databases, as well as results of RT-qPCR (Chu et al., 2020; Zhou et al., 2021).

Under three abiotic stresses, nine tested *ShCDCPs* showed two transcription patterns that were cultivar-dependent and -independent. For instance, *ShCBSD-PB1-5A* and *ShCBSD-PB1-7A-1* were negatively induced by each abiotic stress tested in both cultivars. Rice *OsCBSCBSPB4* (homolog of *ShCBSD-PB1-5A*) can respond to high and low temperature, oxidative and salt stress, whereas overexpression of *OsCBSCBSPB4* in *Escherichia coli* resulted in higher survival rate under salt, oxidative, PEG and high temperature stress, indicating that this gene is likely involved in abiotic stress response and is a potential candidate for producing plants that have enhanced tolerance to multiple abiotic stresses (Kumar et al., 2018). *ShCBS-1D-2* was upregulated in ROC22 but downregulated in MT11-610 under NaCl treatment. However, this gene acted as a negative regulator in both cultivars under PEG6000 and exogenous SA treatments. Overexpression of *OsCBSX4* (a homolog of *ShCBS-1D-2*) in transgenic tobacco was associated with better tolerance to salt, oxidation, and heavy metals, as well as a higher survival rate of plants under stress via reduction of H_2_O_2_ content (Singh et al., 2012). The *Arabidopsis* genome contains six *CBSXs*, which may directly regulate activation of thioredoxin to control levels of intracellular H_2_O_2_ and affect plant growth and development (Ok et al., 2012). A recent study showed that *CBSX3-Trxo-2* (homolog of *ShCBS-1D-2*) regulates ROS generation and plays an important role in regulating plant development and the redox system in *Arabidopsis* (Shin et al., 2020). Other CDCP family genes are also involved in stress responses. For example, overexpression of *GmCBS21* (homolog of *ShTlyc-1D-2*) or *GmCBSDUF3* (homolog of *ShTlyc-1D-2*) enhanced tolerance to low nitrogen levels, drought, and salt stresses in *Arabidopsis* (Hao et al., 2021). *OsCBSX9* (homolog of *ShCBS-4D-2*) and *OsCBSCBS4* (homolog of *ShCBSDCBS-3B-1*) displayed higher expression under drought as well as salinity stress conditions in rice (Tomar et al., 2022).

In general, two expression profiles of duplicated gene pairs in sugarcane responding to various stressors were observed in this study. Three gene pairs with fragment duplication events exhibited functional redundancy or divergent stress responses. Interestingly, the gene pairs *ShCBSD-PB1-5A* and *ShCBSD-PB1-7A-1* and *ShCBSD-PB1-3A* and *ShCBSD-PB1-3C-4* contained similar domains (two CBS domains and one PB1 domain) that shared functional redundancy of two gene pairs responding to various stressors. On the other hand, the gene pair *ShCBS-4C* and *ShCBS-4D-1* contained different domains, i.e., *ShCBS-4C* included two domains (CBS and COG2905) while *ShCBS-4D-1* contained one CBS domain. The two genes *ShCBS-4C* and *ShCBS-4D-1* exhibited functional divergence in response to various stressors and require further investigation. Hughes et al. (2014) demonstrated that 13% of all duplicated genes (homologous gene pairs) underwent enhanced purifying selection to exhibit regulatory neofunctionalization (functional divergence) in *Zea mays*. A similar observation by Tomar et al. (2022) reported that functional divergence of two duplicate gene pairs *OsCBSCBS2* (homolog of *SsCBSDCBS-3D*) and *OsCBSCBS3* (homolog of *SsCBSDCBS-5B*) and *OsCBSCBSPB2* (homolog of *ShCBSD-PB1-5A*) and *OsCBSCBSPB4* (homolog of *ShCBSD-PB1-5A*) are expressed in Oryza species under drought stress.

## Conclusion

A total of 95 *SsCDCP* genes with 87 alleles were systematically identified in the *S. spontaneum* genome (AP85-441) and were classified into eight phylogenetic groups. Gene duplication played an important driving force in the expansion and evolution of *SsCDCP* genes. The RNA-seq dataset and/or RT-qPCR analysis revealed that *ShCDCP* genes displayed different expression patterns in sugarcane under biotic (*Aaa*) and abiotic (drought, salinity, and SA) stresses. *ShCBSD-PB1-5A* and *ShCBSD-PB1-7A-1* served as negative regulators in sugarcane under multiple stress conditions, while *ShCBS-4D-1* and *ShCBSDCBS-5A* played a positive role in sugarcane under *Aaa* infection and in a specific cultivar (ROC22) under abiotic stress conditions (NaCl, PEG6000, and SA). Although functional redundancy and divergence among *ShCDCPs* were present in this study, detailed molecular mechanisms must be further explored in sugarcane in response to multiple stressors using forward (overexpression) and reverse (knockdown) genetics.

## Data availability statement

The datasets presented in this study can be found in online repositories. The names of the repository/repositories and accession number(s) can be found in the article/Supplementary material.

## Author contributions

J-RZ and S-JG: conceptualization and writing—review and editing. J-RZ and JL: writing—original draft preparation. J-RZ, JL, and J-XL: bioinformatics analysis. J-XL, H-MX, and NC: data reduction. Q-NW and S-JG: supervision, funding acquisition, and project administration. All authors have read and agreed to the published version of the manuscript.

## Footnotes

1
https://www.arabidopsis.org/

2
https://www.uniprot.org/

3
http://www.life.illinois.edu/ming/downloads/Spontaneum_genome/

4
https://blast.ncbi.nlm.nih.gov/Blast.cgi

5
http://www.ncbi.nlm.nih.gov/cdd/

6
http://pfam.xfam.org/

7
http://smart.embl.de/

8
https://web.expasy.org/protparam/

9
http://www.csbio.sjtu.edu.cn/bioinf/plant-multi/

10
https://www.evolgenius.info/evolview/

11
http://meme-suite.org/tools/meme

12
http://bioinformatics.psb.ugent.be/webtools/plantcare/html/
