# Supplementary material for: Genome-wide characterization of cys-tathionine-β-synthase domain-containing proteins in sugarcane reveals their role in defense responses under multiple stressors
Source: Front Plant Sci. 2022 Aug 25;13:985653. doi: 10.3389/fpls.2022.985653 (PMC9453547; doi:10.3389/fpls.2022.985653)
Supplement: Supplementary file 1 [file Data_Sheet_1.ZIP › Supplementary Materials-20220801.docx]

**Table S1** Primers used for RT-qPCR assay in this study.

| Target gene | Forward primer (5'-3') | Reverse primer (5'-3') | Tm (^o^C) | Product size (bp) |
| --- | --- | --- | --- | --- |
| *GAPDH* | CACGGCCACTGGAAGCA | TCCTCAGGGTTCCTGATGCC | 58 | 110 |
| *SsCBS-1D-2* | TCGGCAGAGGAGAGGAGGAT | GAACAGGTGGACCAGGAACG | 60 | 128 |
| *SsCBSD-PB1-3C-4* | ACGGACAAGGACATAACCACA | TTTCAACGCCCTCAACAGC | 58 | 289 |
| *SsCBSD-PB1-3A* | TACGGACAAGGACATAACCACAA | TGACTTCACCATGCTCCACAACA | 62 | 185 |
| *SsCBS-4D-1* | CGTGCTGATGTTCTTCGTGT | ACTGATGGCACAGCACATTC | 55 | 124 |
| *SsCBSDCBS-5A* | TGTAGAAGTGGGAGGAGATAAG | TGACAAGTTTCGATGGTTTC | 53 | 158 |
| *SsCBS-5D* | TTGTCCAACCTACCACACCG | AACCCCAATCAACTTCCCAT | 59 | 104 |
| *SsCBSD-PB1-7A-1* | ATGGAACTGTGAATGATGTCGCA | GCAGGAGTAAGGATACCTCGCTA | 61 | 117 |
| *SsCBSD-PB1-5A* | ACGGAATCCTATTTATGTGATGTCT | GCAATAACCTCACCATTCTCTACAA | 59 | 111 |
| *SsCBS-4C* | CTCCAACAAGGACAAGGCCAAGG | CGAGCGAGTCACAATTCCGATCA | 67 | 193 |

**Table S2** The alleles in each *SsCDCP* genes identified in S. spontaneum AP85-441.

| **Group** | **Gene model** | **Alleles** | **Gene ID** | **Gene name** |
| --- | --- | --- | --- | --- |
| D | Sspon.01G0001530 | 3 | Sspon.01G0001530-1A | SsTlyc-1A |
| D |  |  | Sspon.01G0001530-1P | SsTlyc-1D-2 |
| D |  |  | Sspon.01G0001530-2B | SsTlyc-1B-2 |
| F | Sspon.01G0020640 | 3 | Sspon.01G0020640-1A | SsCBS-1A |
| F |  |  | Sspon.01G0020640-2C | SsCBS-1C-1 |
| F |  |  | Sspon.01G0020640-3D | SsCBS-1D-1 |
| C2 | Sspon.01G0025410 | 2 | Sspon.01G0025410-1A | SsCBS-CLC-1A |
| C2 |  |  | Sspon.01G0025410-2B | SsCBS-CLC-1B |
| G | Sspon.01G0033510 | 3 | Sspon.01G0033510-1A | SsCBSD-AMPK1-1A |
| G |  |  | Sspon.01G0033510-1P | SsCBSD-AMPK1-1D-1 |
| G |  |  | Sspon.01G0033510-2D | SsCBSD-AMPK1-1D-2 |
| D | Sspon.01G0044900 | 2 | Sspon.01G0044900-1P | SsTlyc-1D-1 |
| D |  |  | Sspon.01G0044900-2C | SsTlyc-1C-1 |
| D | Sspon.01G0046430 | 1 | Sspon.01G0046430-1B | SsTlyc-1B-1 |
| B | Sspon.01G0057070 | 2 | Sspon.01G0057070-1C | SsCBS-1C-2 |
| B |  |  | Sspon.01G0057070-2D | SsCBS-1D-2 |
| B | Sspon.02G0049550 | 2 | Sspon.02G0049550-1C | SsCBS-2C |
| B |  |  | Sspon.02G0049550-2D | SsCBS-2D |
| A | Sspon.03G0000340 | 2 | Sspon.03G0000340-1A | SsCBSD-PB1-3A |
| A |  |  | Sspon.03G0000340-2C | SsCBSD-PB1-3C-4 |
| H | Sspon.03G0000380 | 3 | Sspon.03G0000380-1A | SsCBSDCBS-3A-3 |
| H |  |  | Sspon.03G0000380-1P | SsCBSDCBS-3D |
| H |  |  | Sspon.03G0000380-2D | SsCBSDCBS-5D-1 |
| F | Sspon.03G0000490 | 4 | Sspon.03G0000490-1A | SsCBS-3A-1 |
| F |  |  | Sspon.03G0000490-1P | SsCBS-3A-2 |
| F |  |  | Sspon.03G0000490-2B | SsCBS-3B-2 |
| F |  |  | Sspon.03G0000490-3D | SsCBS-3D-2 |
| C1 | Sspon.03G0004960 | 1 | Sspon.03G0004960-1A | SsCBS-CLC-3A |
| H | Sspon.03G0010600 | 3 | Sspon.03G0010600-1A | SsCBSDCBS-3A-1 |
| H |  |  | Sspon.03G0010600-1P | SsCBSDCBS-3B-1 |
| H |  |  | Sspon.03G0010600-2B | SsCBSDCBS-3B-2 |
| H | Sspon.03G0012400 | 3 | Sspon.03G0012400-1A | SsCBSDCBS-3A-2 |
| H |  |  | Sspon.03G0012400-2B | SsCBSDCBS-3B-3 |
| H |  |  | Sspon.03G0012400-3C | SsCBSDCBS-3C |
| C2 | Sspon.03G0029010 | 1 | Sspon.03G0029010-3D | SsCBS-CLC-3D |
| B | Sspon.03G0032200 | 3 | Sspon.03G0032200-1B | SsCBS-3B-1 |
| B |  |  | Sspon.03G0032200-2C | SsCBS-3C |
| B |  |  | Sspon.03G0032200-3D | SsCBS-3D-1 |
| A | Sspon.03G0038630 | 3 | Sspon.03G0038630-1C | SsCBSD-PB1-3C-2 |
| A |  |  | Sspon.03G0038630-1P | SsCBSD-PB1-3C-1 |
| A |  |  | Sspon.03G0038630-2D | SsCBSD-PB1-3D-1 |
| C1 | Sspon.04G0004760 | 2 | Sspon.04G0004760-1A | SsCBS-CLC-4A-1 |
| C1 |  |  | Sspon.04G0004760-2B | SsCBS-CLC-4B-1 |
| F | Sspon.04G0007500 | 2 | Sspon.04G0007500-1A | SsCBS-4A-2 |
| F |  |  | Sspon.04G0007500-2D | SsCBS-4D-3 |
| C2 | Sspon.04G0009220 | 6 | Sspon.04G0009220-1A | SsCBS-CLC-4A-3 |
| C2 |  |  | Sspon.04G0009220-1P | SsCBS-CLC-5A |
| C2 |  |  | Sspon.04G0009220-2B | SsCBS-CLC-5B-2 |
| C2 |  |  | Sspon.04G0009220-2P | SsCBS-CLC-5C-2 |
| C2 |  |  | Sspon.04G0009220-3C | SsCBS-CLC-4C |
| C2 |  |  | Sspon.04G0009220-4D | SsCBS-CLC-5D-2 |
| E | Sspon.04G0013500 | 1 | Sspon.04G0013500-2D | SsCBS-SIS-4D |
| C2 | Sspon.04G0016160 | 3 | Sspon.04G0016160-1A | SsCBS-CLC-4A-2 |
| C2 |  |  | Sspon.04G0016160-2B | SsCBS-CLC-4B-2 |
| C2 |  |  | Sspon.04G0016160-3D | SsCBS-CLC-4D |
| F | Sspon.04G0017870 | 3 | Sspon.04G0017870-1A | SsCBS-4A-1 |
| F |  |  | Sspon.04G0017870-2B | SsCBS-4B |
| F |  |  | Sspon.04G0017870-3D | SsCBS-4D-2 |
| D | Sspon.04G0030670 | 2 | Sspon.04G0030670-1C | SsCBS-4C |
| D |  |  | Sspon.04G0030670-1P | SsCBS-4D-1 |
| E | Sspon.04G0034040 | 1 | Sspon.04G0034040-1C | SsCBS-SIS-4C |
| G | Sspon.05G0012300 | 1 | Sspon.05G0012300-2D | SsCBSD-AMPK1-5D |
| H | Sspon.05G0013010 | 4 | Sspon.05G0013010-1A | SsCBSDCBS-5A |
| H |  |  | Sspon.05G0013010-2B | SsCBSDCBS-5B |
| H |  |  | Sspon.05G0013010-3C | SsCBSDCBS-5C |
| H |  |  | Sspon.05G0013010-4D | SsCBSDCBS-5D-2 |
| B | Sspon.05G0014080 | 4 | Sspon.05G0014080-1A | SsCBS-5A |
| B |  |  | Sspon.05G0014080-2B | SsCBS-5B |
| B |  |  | Sspon.05G0014080-3C | SsCBS-5C |
| B |  |  | Sspon.05G0014080-4D | SsCBS-5D |
| A | Sspon.05G0018330 | 3 | Sspon.05G0018330-1A | SsCBSD-PB1-5A |
| A |  |  | Sspon.05G0018330-1P | SsCBSD-PB1-7A-1 |
| A |  |  | Sspon.05G0018330-2C | SsCBSD-PB1-5C |
| A | Sspon.05G0018450 | 1 | Sspon.05G0018450-2D | SsCBSD-PB1-7D-1 |
| C2 | Sspon.05G0022270 | 3 | Sspon.05G0022270-1B | SsCBS-CLC-5B-1 |
| C2 |  |  | Sspon.05G0022270-2C | SsCBS-CLC-5C-1 |
| C2 |  |  | Sspon.05G0022270-3D | SsCBS-CLC-5D-1 |
| C1 | Sspon.06G0000980 | 5 | Sspon.06G0000980-1A | SsCBS-CLC-6A-1 |
| C1 |  |  | Sspon.06G0000980-1P | SsCBS-CLC-6A-2 |
| C1 |  |  | Sspon.06G0000980-2B | SsCBS-CLC-6B |
| C1 |  |  | Sspon.06G0000980-3C | SsCBS-CLC-6C |
| C1 |  |  | Sspon.06G0000980-4D | SsCBS-CLC-6D |
| A | Sspon.06G0025100 | 2 | Sspon.06G0025100-1B | SsCBSD-PB1-6B |
| A |  |  | Sspon.06G0025100-3D | SsCBSD-PB1-6D |
| D | Sspon.07G0010520 | 4 | Sspon.07G0010520-1A | SsTlyc-7A-1 |
| D |  |  | Sspon.07G0010520-1P | SsTlyc-1C-2 |
| D |  |  | Sspon.07G0010520-2B | SsTlyc-7B |
| D |  |  | Sspon.07G0010520-3C | SsTlyc-7C-1 |
| A | Sspon.07G0019280 | 2 | Sspon.07G0019280-1A | SsCBSD-PB1-7A-2 |
| A |  |  | Sspon.07G0019280-2D | SsCBSD-PB1-7D-2 |
| D | Sspon.07G0031220 | 1 | Sspon.07G0031220-1C | SsTlyc-7C-2 |
| C2 | Sspon.08G0019260 | 4 | Sspon.08G0019260-1B | SsCBS-CLC-8B-2 |
| C2 |  |  | Sspon.08G0019260-1T | SsCBS-CLC-8B-1 |
| C2 |  |  | Sspon.08G0019260-2C | SsCBS-CLC-8C |
| C2 |  |  | Sspon.08G0019260-3D | SsCBS-CLC-8D |

**Table S3** Physico-chemical properties of the *SsCDCP* genes identified in *S. spontaneum* AP85-441.

| **Group** | **Gene ID** | **Gene name** | **Genome location** | **Amino acids (aa)** | **Mw (Da)** | **pI** | **Subcellular localization** | **Exons** | **5’UTR** | **CDS** | **3’UTR** |
| --- | --- | --- | --- | --- | --- | --- | --- | --- | --- | --- | --- |
| A | Sspon.03G0000340-1A | SsCBSD-PB1-3A | Chr3A:950856-954681 | 586 | 63571.14 | 8.78 | Mitochondrial | 13 | 0 | 13 | 0 |
| A | Sspon.03G0038630-1P | SsCBSD-PB1-3C-1 | Chr3C:6783943-6788657 | 453 | 49409.31 | 5.89 | Cytoplasmic | 12 | 0 | 12 | 1 |
| A | Sspon.03G0038630-1C | SsCBSD-PB1-3C-2 | Chr3C:6649348-6653312 | 495 | 52949.10 | 7.21 | Nuclear | 13 | 0 | 13 | 0 |
| A | Sspon.03G0000340-2C | SsCBSD-PB1-3C-4 | Chr3C:11209466-11214322 | 439 | 47260.25 | 9.15 | Mitochondrial | 12 | 1 | 12 | 0 |
| A | Sspon.03G0038630-2D | SsCBSD-PB1-3D-1 | Chr3D:1979176-1983369 | 540 | 58596.04 | 6.29 | Chloroplast | 14 | 0 | 14 | 0 |
| A | Sspon.05G0018330-1A | SsCBSD-PB1-5A | Chr5A:77379467-77384533 | 544 | 58391.77 | 6.08 | Mitochondrial | 14 | 1 | 14 | 1 |
| A | Sspon.05G0018330-2C | SsCBSD-PB1-5C | Chr5C:75979535-75984473 | 482 | 51902.17 | 6.24 | Mitochondrial | 12 | 0 | 12 | 1 |
| A | Sspon.06G0025100-1B | SsCBSD-PB1-6B | Chr6B:61501769-61504535 | 486 | 52602.55 | 5.67 | Chloroplast | 12 | 0 | 12 | 0 |
| A | Sspon.06G0025100-3D | SsCBSD-PB1-6D | Chr6D:57633598-57636348 | 486 | 52406.31 | 5.57 | Chloroplast | 12 | 0 | 12 | 0 |
| A | Sspon.05G0018330-1P | SsCBSD-PB1-7A-1 | Chr7A:70466521-70471906 | 497 | 53629.08 | 8.44 | Nuclear | 13 | 0 | 13 | 0 |
| A | Sspon.07G0019280-1A | SsCBSD-PB1-7A-2 | Chr7A:70489726-70499427 | 170 | 19152.65 | 4.90 | Cytoplasmic | 6 | 0 | 6 | 0 |
| A | Sspon.05G0018450-2D | SsCBSD-PB1-7D-1 | Chr7D:71847149-71849449 | 212 | 22392.60 | 9.77 | Chloroplast | 5 | 1 | 5 | 0 |
| A | Sspon.07G0019280-2D | SsCBSD-PB1-7D-2 | Chr7D:71855930-71857716 | 282 | 30693.97 | 4.84 | Cytoplasmic | 9 | 0 | 9 | 0 |
| B | Sspon.01G0057070-1C | SsCBS-1C-2 | Chr1C:96366142-96371018 | 273 | 29898.43 | 10.27 | Chloroplast | 8 | 0 | 8 | 1 |
| B | Sspon.01G0057070-2D | SsCBS-1D-2 | Chr1D:93023095-93026874 | 182 | 19996.75 | 5.93 | Cytoplasmic | 6 | 0 | 6 | 1 |
| B | Sspon.02G0049550-1C | SsCBS-2C | Chr2C:40556327-40574638 | 615 | 68177.17 | 8.97 | Chloroplast | 12 | 1 | 12 | 0 |
| B | Sspon.02G0049550-2D | SsCBS-2D | Chr2D:31583987-31591218 | 318 | 34798.48 | 8.47 | Chloroplast | 6 | 0 | 6 | 1 |
| B | Sspon.03G0032200-1B | SsCBS-3B-1 | Chr3B:35685807-35687530 | 192 | 21338.53 | 6.11 | Mitochondrial | 5 | 0 | 5 | 0 |
| B | Sspon.03G0032200-2C | SsCBS-3C | Chr3C:44083407-44085174 | 197 | 21964.45 | 6.84 | Mitochondrial | 5 | 1 | 5 | 0 |
| B | Sspon.03G0032200-3D | SsCBS-3D-1 | Chr3D:33645068-33649162 | 249 | 27354.51 | 6.30 | Cytoplasmic | 7 | 0 | 7 | 0 |
| B | Sspon.05G0014080-1A | SsCBS-5A | Chr5A:51149053-51152598 | 195 | 20964.93 | 10.68 | Mitochondrial | 5 | 1 | 5 | 1 |
| B | Sspon.05G0014080-2B | SsCBS-5B | Chr5B:51947840-51951340 | 216 | 23387.66 | 9.29 | Mitochondrial | 7 | 1 | 7 | 0 |
| B | Sspon.05G0014080-3C | SsCBS-5C | Chr5C:53980855-53983953 | 231 | 24787.36 | 9.87 | Mitochondrial | 7 | 1 | 7 | 1 |
| B | Sspon.05G0014080-4D | SsCBS-5D | Chr5D:50411266-50414215 | 233 | 24986.56 | 9.75 | Chloroplast | 7 | 0 | 7 | 1 |
| C1 | Sspon.03G0004960-1A | SsCBS-CLC-3A | Chr3A:14161294-14166454 | 725 | 77676.59 | 6.32 | Plasma membrane | 8 | 0 | 8 | 0 |
| C1 | Sspon.04G0004760-1A | SsCBS-CLC-4A-1 | Chr4A:13880701-13890475 | 803 | 85435.01 | 7.57 | Plasma membrane | 9 | 0 | 9 | 0 |
| C1 | Sspon.04G0004760-2B | SsCBS-CLC-4B-1 | Chr4B:9363218-9367868 | 771 | 82097.46 | 6.98 | Plasma membrane | 9 | 0 | 9 | 0 |
| C1 | Sspon.06G0000980-1A | SsCBS-CLC-6A-1 | Chr6A:3410886-3416325 | 720 | 76416.33 | 6.06 | Plasma membrane | 8 | 1 | 8 | 0 |
| C1 | Sspon.06G0000980-1P | SsCBS-CLC-6A-2 | Chr6A:3428183-3430110 | 252 | 28280.62 | 5.44 | Nuclear | 4 | 0 | 4 | 1 |
| C1 | Sspon.06G0000980-2B | SsCBS-CLC-6B | Chr6B:1857745-1863554 | 752 | 79978.76 | 6.07 | Plasma membrane | 9 | 0 | 9 | 1 |
| C1 | Sspon.06G0000980-3C | SsCBS-CLC-6C | Chr6C:1472254-1476528 | 630 | 67823.27 | 6.35 | Plasma membrane | 8 | 0 | 8 | 1 |
| C1 | Sspon.06G0000980-4D | SsCBS-CLC-6D | Chr6D:987877-993306 | 676 | 71747.65 | 5.97 | Plasma membrane | 9 | 1 | 9 | 0 |
| C2 | Sspon.01G0025410-1A | SsCBS-CLC-1A | Chr1A:89936007-89948321 | 796 | 87415.45 | 8.39 | Plasma membrane | 23 | 1 | 23 | 1 |
| C2 | Sspon.01G0025410-2B | SsCBS-CLC-1B | Chr1B:100534749-100551243 | 829 | 91618.21 | 8.78 | Plasma membrane | 21 | 0 | 21 | 0 |
| C2 | Sspon.03G0029010-3D | SsCBS-CLC-3D | Chr3D:11710398-11713746 | 730 | 77956.21 | 8.49 | Plasma membrane | 6 | 0 | 6 | 0 |
| C2 | Sspon.04G0009220-1A | SsCBS-CLC-4A-3 | Chr4A:26748523-26754610 | 716 | 76980.46 | 6.83 | Plasma membrane | 6 | 1 | 6 | 0 |
| C2 | Sspon.04G0016160-1A | SsCBS-CLC-4A-2 | Chr4A:59313850-59318117 | 693 | 75602.81 | 9.65 | Plasma membrane | 5 | 0 | 5 | 1 |
| C2 | Sspon.04G0016160-2B | SsCBS-CLC-4B-2 | Chr4B:63286521-63293860 | 796 | 86566.97 | 8.89 | Plasma membrane | 6 | 0 | 6 | 0 |
| C2 | Sspon.04G0009220-3C | SsCBS-CLC-4C | Chr4C:29569822-29575294 | 750 | 80768.70 | 6.98 | Plasma membrane | 6 | 1 | 6 | 1 |
| C2 | Sspon.04G0016160-3D | SsCBS-CLC-4D | Chr4D:68511472-68512918 | 410 | 45212.91 | 10.67 | Mitochondrial | 2 | 0 | 2 | 1 |
| C2 | Sspon.04G0009220-1P | SsCBS-CLC-5A | Chr5A:30392963-30398074 | 689 | 74384.32 | 6.65 | Plasma membrane | 6 | 0 | 6 | 1 |
| C2 | Sspon.05G0022270-1B | SsCBS-CLC-5B-1 | Chr5B:3013071-3018342 | 815 | 88503.98 | 8.58 | Plasma membrane | 7 | 1 | 7 | 1 |
| C2 | Sspon.04G0009220-2B | SsCBS-CLC-5B-2 | Chr5B:25306858-25320780 | 723 | 78294.05 | 5.91 | Plasma membrane | 7 | 1 | 7 | 0 |
| C2 | Sspon.05G0022270-2C | SsCBS-CLC-5C-1 | Chr5C:7213191-7218416 | 815 | 88482.97 | 8.51 | Plasma membrane | 7 | 0 | 7 | 1 |
| C2 | Sspon.04G0009220-2P | SsCBS-CLC-5C-2 | Chr5C:21948690-21953329 | 600 | 64575.45 | 6.09 | Plasma membrane | 6 | 0 | 6 | 0 |
| C2 | Sspon.05G0022270-3D | SsCBS-CLC-5D-1 | Chr5D:7070804-7075964 | 814 | 88422.87 | 8.58 | Plasma membrane | 7 | 1 | 7 | 1 |
| C2 | Sspon.04G0009220-4D | SsCBS-CLC-5D-2 | Chr5D:32974332-32979597 | 730 | 78537.23 | 6.20 | Plasma membrane | 6 | 1 | 6 | 1 |
| C2 | Sspon.08G0019260-1T | SsCBS-CLC-8B-1 | Chr8B:11062146-11065080 | 754 | 82903.85 | 9.16 | Plasma membrane | 4 | 0 | 4 | 0 |
| C2 | Sspon.08G0019260-1B | SsCBS-CLC-8B-2 | Chr8B:11030197-11048276 | 778 | 85079.90 | 9.16 | Plasma membrane | 6 | 0 | 6 | 0 |
| C2 | Sspon.08G0019260-2C | SsCBS-CLC-8C | Chr8C:13572046-13574934 | 754 | 82907.84 | 9.16 | Plasma membrane | 4 | 0 | 4 | 0 |
| C2 | Sspon.08G0019260-3D | SsCBS-CLC-8D | Chr8D:10717750-10723768 | 801 | 87858.29 | 6.38 | Plasma membrane | 5 | 1 | 5 | 0 |
| D | Sspon.04G0030670-1C | SsCBS-4C | Chr4C:2120453-2121918 | 195 | 21206.26 | 4.91 | Chloroplast | 5 | 1 | 5 | 1 |
| D | Sspon.04G0030670-1P | SsCBS-4D-1 | Chr4D:3200280-3201750 | 217 | 23513.88 | 5.26 | Chloroplast | 5 | 1 | 5 | 0 |
| D | Sspon.01G0001530-1A | SsTlyc-1A | Chr1A:4508881-4512585 | 437 | 47538.47 | 6.93 | Plasma membrane | 11 | 0 | 11 | 0 |
| D | Sspon.01G0046430-1B | SsTlyc-1B-1 | Chr1B:97420046-97426174 | 407 | 45671.59 | 5.48 | Cytoplasmic | 11 | 1 | 11 | 1 |
| D | Sspon.01G0001530-2B | SsTlyc-1B-2 | Chr1B:3326084-3330778 | 472 | 51052.99 | 6.40 | Plasma membrane | 10 | 1 | 10 | 0 |
| D | Sspon.01G0044900-2C | SsTlyc-1C-1 | Chr1C:53701363-53706165 | 332 | 37549.12 | 4.54 | Cytoplasmic | 8 | 0 | 8 | 1 |
| D | Sspon.07G0010520-1P | SsTlyc-1C-2 | Chr1C:23728896-23741536 | 490 | 52987.10 | 5.32 | Plasma membrane | 13 | 0 | 13 | 1 |
| D | Sspon.01G0044900-1P | SsTlyc-1D-1 | Chr1D:80389023-80395241 | 331 | 37425.02 | 4.54 | Cytoplasmic | 8 | 1 | 8 | 1 |
| D | Sspon.01G0001530-1P | SsTlyc-1D-2 | Chr1D:1109681-1119374 | 490 | 53335.42 | 6.79 | Plasma membrane | 11 | 0 | 11 | 1 |
| D | Sspon.07G0010520-1A | SsTlyc-7A-1 | Chr7A:33830809-33844089 | 534 | 57006.66 | 5.67 | Plasma membrane | 15 | 1 | 15 | 0 |
| D | Sspon.07G0010520-2B | SsTlyc-7B | Chr7B:21689851-21695993 | 238 | 25642.73 | 5.33 | Cytoplasmic | 8 | 0 | 8 | 0 |
| D | Sspon.07G0010520-3C | SsTlyc-7C-1 | Chr7C:19241640-19249931 | 519 | 55372.88 | 5.63 | Plasma membrane | 13 | 1 | 13 | 1 |
| D | Sspon.07G0031220-1C | SsTlyc-7C-2 | Chr7C:19272324-19280972 | 468 | 49898.35 | 5.11 | Plasma membrane | 11 | 1 | 11 | 1 |
| E | Sspon.04G0034040-1C | SsCBS-SIS-4C | Chr4C:70511580-70532370 | 1367 | 147232.08 | 5.56 | Plasma membrane | 8 | 0 | 8 | 0 |
| E | Sspon.04G0013500-2D | SsCBS-SIS-4D | Chr4D:73612402-73617851 | 341 | 35786.48 | 5.68 | Chloroplast | 4 | 1 | 4 | 0 |
| F | Sspon.01G0020640-1A | SsCBS-1A | Chr1A:76605401-76607427 | 377 | 39021.73 | 5.54 | Chloroplast | 2 | 1 | 2 | 0 |
| F | Sspon.01G0020640-2C | SsCBS-1C-1 | Chr1C:77631499-77633678 | 380 | 39206.91 | 5.54 | Chloroplast | 2 | 1 | 2 | 1 |
| F | Sspon.01G0020640-3D | SsCBS-1D-1 | Chr1D:74224549-74226381 | 380 | 39261.01 | 5.54 | Chloroplast | 2 | 0 | 2 | 0 |
| F | Sspon.03G0000490-1A | SsCBS-3A-1 | Chr3A:1449458-1449967 | 169 | 17138.29 | 5.52 | Chloroplast | 1 | 0 | 1 | 0 |
| F | Sspon.03G0000490-1P | SsCBS-3A-2 | Chr3A:1541106-1546501 | 410 | 43660.84 | 7.04 | Chloroplast | 2 | 1 | 2 | 1 |
| F | Sspon.03G0000490-2B | SsCBS-3B-2 | Chr3B:8096871-8100721 | 397 | 42279.48 | 7.68 | Chloroplast | 4 | 0 | 4 | 0 |
| F | Sspon.03G0000490-3D | SsCBS-3D-2 | Chr3D:7049042-7053163 | 410 | 43674.86 | 7.04 | Chloroplast | 2 | 1 | 2 | 0 |
| F | Sspon.04G0017870-1A | SsCBS-4A-1 | Chr4A:64848605-64850091 | 431 | 45492.48 | 5.19 | Chloroplast | 3 | 0 | 3 | 0 |
| F | Sspon.04G0007500-1A | SsCBS-4A-2 | Chr4A:21264355-21271826 | 396 | 41018.89 | 5.89 | Chloroplast | 3 | 0 | 3 | 0 |
| F | Sspon.04G0017870-2B | SsCBS-4B | Chr4B:68463430-68464905 | 428 | 45225.16 | 5.24 | Chloroplast | 3 | 0 | 3 | 0 |
| F | Sspon.04G0017870-3D | SsCBS-4D-2 | Chr4D:73518616-73520106 | 431 | 45384.29 | 5.24 | Chloroplast | 3 | 0 | 3 | 0 |
| F | Sspon.04G0007500-2D | SsCBS-4D-3 | Chr4D:22457464-22458855 | 417 | 43046.32 | 5.99 | Chloroplast | 2 | 0 | 2 | 0 |
| G | Sspon.01G0033510-1A | SsCBSD-AMPK1-1A | Chr1A:112763506-112771928 | 496 | 54882.53 | 6.25 | Nuclear | 13 | 1 | 13 | 1 |
| G | Sspon.01G0033510-1P | SsCBSD-AMPK1-1D-1 | Chr1D:111200463-111207212 | 496 | 54930.56 | 6.25 | Plasma membrane | 13 | 1 | 13 | 1 |
| G | Sspon.01G0033510-2D | SsCBSD-AMPK1-1D-2 | Chr1D:114615509-114623114 | 438 | 48641.19 | 5.88 | Plasma membrane | 12 | 1 | 12 | 1 |
| G | Sspon.05G0012300-2D | SsCBSD-AMPK1-5D | Chr5D:38804998-38813638 | 447 | 49897.62 | 5.66 | Plasma membrane | 13 | 1 | 13 | 1 |
| H | Sspon.03G0010600-1A | SsCBSDCBS-3A-1 | Chr3A:28809032-28811676 | 419 | 45326.23 | 5.23 | Chloroplast | 7 | 1 | 7 | 1 |
| H | Sspon.03G0012400-1A | SsCBSDCBS-3A-2 | Chr3A:34180989-34188669 | 541 | 58037.63 | 6.07 | Chloroplast | 6 | 0 | 6 | 1 |
| H | Sspon.03G0000380-1A | SsCBSDCBS-3A-3 | Chr3A:1191539-1193202 | 423 | 46166.59 | 5.88 | Cytoplasmic | 5 | 0 | 5 | 0 |
| H | Sspon.03G0010600-1P | SsCBSDCBS-3B-1 | Chr3B:35852684-35855407 | 428 | 46291.40 | 5.12 | Chloroplast | 7 | 0 | 7 | 1 |
| H | Sspon.03G0010600-2B | SsCBSDCBS-3B-2 | Chr3B:35819173-35821567 | 413 | 44638.68 | 5.21 | Chloroplast | 8 | 0 | 8 | 1 |
| H | Sspon.03G0012400-2B | SsCBSDCBS-3B-3 | Chr3B:44339474-44341974 | 451 | 48198.48 | 4.89 | Cytoplasmic | 5 | 0 | 5 | 1 |
| H | Sspon.03G0012400-3C | SsCBSDCBS-3C | Chr3C:50866278-50868448 | 347 | 38115.96 | 4.97 | Cytoplasmic | 6 | 0 | 6 | 1 |
| H | Sspon.03G0000380-1P | SsCBSDCBS-3D | Chr3D:6710487-6712129 | 436 | 47477.30 | 5.55 | Chloroplast | 3 | 0 | 3 | 0 |
| H | Sspon.05G0013010-1A | SsCBSDCBS-5A | Chr5A:41113974-41119181 | 423 | 46654.24 | 5.42 | Cytoplasmic | 6 | 1 | 6 | 0 |
| H | Sspon.05G0013010-2B | SsCBSDCBS-5B | Chr5B:36114397-36118461 | 423 | 46636.21 | 5.42 | Cytoplasmic | 5 | 0 | 22 | 0 |
| H | Sspon.05G0013010-3C | SsCBSDCBS-5C | Chr5C:34942561-34946975 | 354 | 39538.60 | 5.54 | Cytoplasmic | 5 | 1 | 5 | 1 |
| H | Sspon.03G0000380-2D | SsCBSDCBS-5D-1 | Chr5D:33833203-33834951 | 416 | 45380.84 | 5.48 | Chloroplast | 7 | 0 | 7 | 0 |
| H | Sspon.05G0013010-4D | SsCBSDCBS-5D-2 | Chr5D:42446931-42452776 | 423 | 46670.20 | 5.34 | Cytoplasmic | 7 | 1 | 7 | 0 |

**Table S4** The *cis*-regulatory elements predicted in *SsCDCP* genes

| **Group** | **gene name** | **ABRE** | **AuxRR-core** | **CGTCA**  **motif** | **TCA-element** | **TGACG motif** | **TGA-element** | **P box** | **ERE** | **GARE-motif** | **ARE** | **DRE core** | **WRE3** |
| --- | --- | --- | --- | --- | --- | --- | --- | --- | --- | --- | --- | --- | --- |
| A | SsCBSD-PB1-3A | 2 | 0 | 2 | 1 | 2 | 1 | 1 | 0 | 0 | 1 | 1 | 4 |
| A | SsCBSD-PB1-3C-1 | 2 | 0 | 11 | 1 | 11 | 1 | 0 | 0 | 2 | 3 | 1 | 1 |
| A | SsCBSD-PB1-3C-2 | 1 | 0 | 0 | 0 | 0 | 1 | 0 | 0 | 0 | 1 | 0 | 2 |
| A | SsCBSD-PB1-3C-4 | 6 | 0 | 2 | 0 | 2 | 2 | 1 | 0 | 1 | 1 | 0 | 4 |
| A | SsCBSD-PB1-3D-1 | 2 | 0 | 0 | 0 | 0 | 2 | 0 | 0 | 0 | 2 | 0 | 2 |
| A | SsCBSD-PB1-5A | 6 | 0 | 5 | 2 | 5 | 1 | 1 | 0 | 2 | 1 | 1 | 3 |
| A | SsCBSD-PB1-5C | 5 | 1 | 3 | 1 | 3 | 1 | 0 | 0 | 1 | 0 | 1 | 2 |
| A | SsCBSD-PB1-6B | 3 | 0 | 1 | 1 | 1 | 0 | 1 | 4 | 0 | 3 | 0 | 5 |
| A | SsCBSD-PB1-6D | 3 | 0 | 1 | 1 | 1 | 0 | 1 | 3 | 0 | 2 | 0 | 5 |
| A | SsCBSD-PB1-7A-1 | 0 | 0 | 1 | 0 | 1 | 1 | 0 | 0 | 0 | 6 | 0 | 1 |
| A | SsCBSD-PB1-7A-2 | 3 | 1 | 1 | 2 | 1 | 2 | 1 | 0 | 1 | 2 | 0 | 0 |
| A | SsCBSD-PB1-7D-1 | 4 | 0 | 1 | 1 | 1 | 1 | 1 | 0 | 0 | 3 | 0 | 2 |
| A | SsCBSD-PB1-7D-2 | 3 | 0 | 4 | 0 | 4 | 0 | 0 | 1 | 0 | 1 | 0 | 1 |
| B | SsCBS-1C-2 | 1 | 0 | 1 | 0 | 1 | 1 | 1 | 1 | 0 | 0 | 3 | 1 |
| B | SsCBS-1D-2 | 0 | 0 | 0 | 2 | 0 | 0 | 1 | 1 | 0 | 4 | 0 | 1 |
| B | SsCBS-2C | 1 | 0 | 2 | 0 | 2 | 0 | 0 | 1 | 0 | 2 | 2 | 2 |
| B | SsCBS-2D | 1 | 0 | 0 | 0 | 0 | 0 | 0 | 1 | 0 | 1 | 0 | 0 |
| B | SsCBS-3B-1 | 4 | 0 | 3 | 3 | 3 | 0 | 0 | 1 | 0 | 3 | 0 | 0 |
| B | SsCBS-3C | 4 | 0 | 1 | 3 | 1 | 0 | 0 | 1 | 0 | 2 | 1 | 0 |
| B | SsCBS-3D-1 | 4 | 0 | 1 | 3 | 1 | 0 | 0 | 3 | 0 | 3 | 0 | 1 |
| B | SsCBS-5A | 0 | 0 | 0 | 0 | 0 | 0 | 0 | 0 | 0 | 0 | 0 | 1 |
| B | SsCBS-5B | 0 | 0 | 0 | 0 | 0 | 0 | 0 | 0 | 0 | 0 | 0 | 0 |
| B | SsCBS-5C | 0 | 0 | 0 | 0 | 0 | 0 | 0 | 0 | 0 | 0 | 0 | 2 |
| B | SsCBS-5D | 9 | 0 | 2 | 0 | 2 | 0 | 0 | 0 | 0 | 0 | 1 | 2 |
| B | SsCBS-CLC-3A | 8 | 0 | 6 | 1 | 6 | 0 | 0 | 0 | 0 | 0 | 2 | 2 |
| C1 | SsCBS-CLC-4A-1 | 4 | 0 | 4 | 1 | 4 | 0 | 0 | 2 | 0 | 1 | 1 | 0 |
| C1 | SsCBS-CLC-4B-1 | 13 | 0 | 4 | 0 | 4 | 1 | 1 | 2 | 1 | 2 | 1 | 0 |
| C1 | SsCBS-CLC-6A-1 | 3 | 0 | 5 | 0 | 5 | 5 | 1 | 0 | 1 | 2 | 5 | 0 |
| C1 | SsCBS-CLC-6A-2 | 2 | 0 | 2 | 1 | 2 | 0 | 0 | 0 | 0 | 1 | 0 | 2 |
| C1 | SsCBS-CLC-6B | 0 | 1 | 1 | 1 | 1 | 3 | 1 | 0 | 0 | 3 | 2 | 4 |
| C1 | SsCBS-CLC-6C | 4 | 0 | 2 | 0 | 2 | 3 | 1 | 0 | 0 | 1 | 0 | 1 |
| C1 | SsCBS-CLC-6D | 0 | 1 | 2 | 1 | 2 | 3 | 1 | 0 | 0 | 3 | 2 | 3 |
| C1 | SsCBS-CLC-1A | 3 | 0 | 5 | 0 | 5 | 0 | 0 | 0 | 0 | 1 | 0 | 0 |
| C2 | SsCBS-CLC-1B | 4 | 0 | 4 | 0 | 4 | 0 | 0 | 0 | 0 | 1 | 0 | 0 |
| C2 | SsCBS-CLC-3D | 4 | 0 | 1 | 0 | 1 | 2 | 0 | 0 | 0 | 1 | 0 | 0 |
| C2 | SsCBS-CLC-4A-3 | 7 | 0 | 6 | 2 | 6 | 1 | 0 | 1 | 0 | 1 | 1 | 1 |
| C2 | SsCBS-CLC-4A-2 | 1 | 0 | 3 | 0 | 3 | 0 | 1 | 0 | 0 | 0 | 2 | 0 |
| C2 | SsCBS-CLC-4B-2 | 3 | 2 | 1 | 0 | 1 | 0 | 0 | 0 | 0 | 1 | 0 | 3 |
| C2 | SsCBS-CLC-4C | 8 | 0 | 6 | 3 | 6 | 1 | 0 | 1 | 0 | 1 | 1 | 1 |
| C2 | SsCBS-CLC-4D | 3 | 0 | 5 | 0 | 5 | 1 | 2 | 0 | 0 | 2 | 2 | 2 |
| C2 | SsCBS-CLC-5A | 8 | 0 | 3 | 0 | 3 | 0 | 0 | 0 | 1 | 2 | 1 | 0 |
| C2 | SsCBS-CLC-5B-1 | 3 | 0 | 3 | 1 | 3 | 2 | 1 | 0 | 0 | 1 | 0 | 1 |
| C2 | SsCBS-CLC-5B-2 | 10 | 0 | 2 | 0 | 2 | 0 | 0 | 0 | 1 | 3 | 1 | 0 |
| C2 | SsCBS-CLC-5C-1 | 6 | 0 | 4 | 1 | 4 | 0 | 0 | 0 | 0 | 0 | 0 | 1 |
| C2 | SsCBS-CLC-5C-2 | 10 | 0 | 4 | 0 | 4 | 0 | 1 | 0 | 1 | 3 | 1 | 0 |
| C2 | SsCBS-CLC-5D-1 | 3 | 0 | 3 | 1 | 3 | 0 | 1 | 0 | 0 | 0 | 0 | 1 |
| C2 | SsCBS-CLC-5D-2 | 11 | 0 | 2 | 1 | 2 | 0 | 0 | 1 | 1 | 2 | 1 | 0 |
| C2 | SsCBS-CLC-8B-1 | 0 | 0 | 2 | 0 | 2 | 1 | 3 | 1 | 0 | 0 | 2 | 1 |
| C2 | SsCBS-CLC-8B-2 | 1 | 0 | 2 | 0 | 2 | 1 | 3 | 1 | 0 | 0 | 2 | 1 |
| C2 | SsCBS-CLC-8C | 0 | 0 | 2 | 0 | 2 | 1 | 3 | 2 | 0 | 0 | 2 | 1 |
| C2 | SsCBS-CLC-8D | 1 | 0 | 0 | 0 | 0 | 0 | 2 | 2 | 0 | 2 | 1 | 1 |
| C2 | SsCBS-4C | 8 | 1 | 2 | 0 | 2 | 1 | 0 | 0 | 0 | 7 | 2 | 2 |
| D | SsCBS-4D-1 | 8 | 1 | 3 | 0 | 3 | 1 | 0 | 0 | 0 | 5 | 2 | 2 |
| D | SsTlyc-1A | 7 | 0 | 4 | 0 | 4 | 1 | 0 | 0 | 0 | 1 | 1 | 0 |
| D | SsTlyc-1B-1 | 7 | 0 | 10 | 0 | 10 | 0 | 0 | 0 | 0 | 3 | 3 | 3 |
| D | SsTlyc-1B-2 | 5 | 0 | 2 | 0 | 2 | 1 | 0 | 0 | 0 | 1 | 0 | 0 |
| D | SsTlyc-1C-1 | 5 | 0 | 3 | 1 | 3 | 0 | 1 | 1 | 0 | 1 | 0 | 0 |
| D | SsTlyc-1C-2 | 5 | 0 | 3 | 0 | 3 | 2 | 1 | 0 | 0 | 1 | 0 | 0 |
| D | SsTlyc-1D-1 | 6 | 0 | 3 | 1 | 3 | 1 | 1 | 0 | 0 | 0 | 1 | 3 |
| D | SsTlyc-1D-2 | 7 | 0 | 2 | 0 | 2 | 0 | 0 | 0 | 0 | 1 | 0 | 0 |
| D | SsTlyc-7A-1 | 5 | 0 | 2 | 0 | 2 | 2 | 1 | 1 | 0 | 2 | 0 | 1 |
| D | SsTlyc-7B | 4 | 0 | 3 | 0 | 3 | 1 | 0 | 0 | 0 | 1 | 0 | 1 |
| D | SsTlyc-7C-1 | 5 | 1 | 0 | 0 | 0 | 1 | 2 | 0 | 0 | 0 | 0 | 1 |
| D | SsTlyc-7C-2 | 5 | 0 | 6 | 0 | 6 | 2 | 0 | 1 | 0 | 1 | 0 | 0 |
| D | SsCBS-SIS-4C | 6 | 1 | 1 | 0 | 1 | 0 | 0 | 0 | 0 | 1 | 1 | 1 |
| E | SsCBS-SIS-4D | 6 | 1 | 7 | 0 | 7 | 1 | 1 | 1 | 0 | 0 | 0 | 4 |
| E | SsCBS-1A | 1 | 0 | 1 | 1 | 1 | 1 | 0 | 0 | 0 | 4 | 0 | 0 |
| F | SsCBS-1C-1 | 7 | 0 | 3 | 0 | 3 | 0 | 0 | 0 | 0 | 1 | 0 | 0 |
| F | SsCBS-1D-1 | 6 | 0 | 3 | 0 | 3 | 0 | 0 | 0 | 0 | 2 | 0 | 1 |
| F | SsCBS-3A-1 | 4 | 1 | 3 | 0 | 3 | 1 | 0 | 0 | 0 | 1 | 0 | 1 |
| F | SsCBS-3A-2 | 4 | 1 | 3 | 0 | 3 | 1 | 0 | 0 | 0 | 1 | 1 | 2 |
| F | SsCBS-3B-2 | 2 | 0 | 6 | 0 | 6 | 1 | 0 | 0 | 1 | 0 | 0 | 2 |
| F | SsCBS-3D-2 | 2 | 2 | 4 | 0 | 4 | 1 | 0 | 0 | 0 | 3 | 0 | 2 |
| F | SsCBS-4A-1 | 7 | 0 | 8 | 1 | 8 | 0 | 1 | 0 | 0 | 1 | 1 | 1 |
| F | SsCBS-4A-2 | 9 | 0 | 6 | 1 | 6 | 0 | 0 | 0 | 0 | 0 | 3 | 1 |
| F | SsCBS-4B | 6 | 0 | 7 | 1 | 7 | 0 | 1 | 0 | 0 | 2 | 1 | 2 |
| F | SsCBS-4D-2 | 7 | 0 | 9 | 1 | 9 | 0 | 1 | 0 | 0 | 2 | 1 | 1 |
| F | SsCBS-4D-3 | 10 | 0 | 6 | 0 | 6 | 0 | 0 | 0 | 0 | 0 | 2 | 0 |
| F | SsCBSD-AMPK1-1A | 10 | 0 | 0 | 2 | 0 | 0 | 1 | 0 | 0 | 0 | 0 | 2 |
| G | SsCBSD-AMPK1-1D-1 | 7 | 0 | 0 | 2 | 0 | 0 | 1 | 0 | 0 | 2 | 0 | 1 |
| G | SsCBSD-AMPK1-1D-2 | 14 | 0 | 0 | 0 | 0 | 1 | 1 | 0 | 1 | 0 | 1 | 0 |
| G | SsCBSD-AMPK1-5D | 2 | 0 | 1 | 0 | 1 | 0 | 0 | 1 | 0 | 3 | 1 | 0 |
| G | SsCBSDCBS-3A-1 | 5 | 0 | 2 | 0 | 2 | 0 | 0 | 0 | 0 | 0 | 0 | 2 |
| H | SsCBSDCBS-3A-2 | 3 | 0 | 1 | 2 | 1 | 0 | 0 | 0 | 0 | 2 | 0 | 4 |
| H | SsCBSDCBS-3A-3 | 0 | 1 | 2 | 0 | 2 | 2 | 0 | 0 | 0 | 0 | 0 | 1 |
| H | SsCBSDCBS-3B-1 | 7 | 0 | 2 | 0 | 2 | 0 | 0 | 0 | 0 | 0 | 1 | 2 |
| H | SsCBSDCBS-3B-2 | 6 | 0 | 2 | 0 | 3 | 0 | 0 | 0 | 0 | 0 | 1 | 3 |
| H | SsCBSDCBS-3B-3 | 1 | 0 | 2 | 0 | 2 | 0 | 1 | 0 | 0 | 3 | 1 | 1 |
| H | SsCBSDCBS-3C | 1 | 0 | 0 | 0 | 0 | 0 | 1 | 0 | 0 | 0 | 1 | 1 |
| H | SsCBSDCBS-3D | 3 | 1 | 0 | 2 | 0 | 0 | 0 | 1 | 0 | 2 | 0 | 2 |
| H | SsCBSDCBS-5A | 7 | 2 | 3 | 1 | 3 | 1 | 1 | 0 | 0 | 2 | 2 | 1 |
| H | SsCBSDCBS-5B | 8 | 0 | 1 | 0 | 1 | 0 | 0 | 0 | 0 | 2 | 1 | 2 |
| H | SsCBSDCBS-5C | 7 | 1 | 3 | 0 | 3 | 0 | 1 | 0 | 0 | 1 | 1 | 2 |
| H | SsCBSDCBS-5D-1 | 7 | 1 | 1 | 0 | 1 | 1 | 0 | 0 | 1 | 1 | 1 | 4 |
| H | SsCBSDCBS-5D-2 | 0 | 2 | 4 | 1 | 4 | 1 | 0 | 0 | 2 | 4 | 1 | 2 |

Continued table S4

| **Group** | **gene name** | **WUN-motif** | **TC-rich repeats** | **STRE** | **GC motif** | **LTR** | **MBS** | **MRE** | **MYB** | **MYB recognition site** | **Myb-binding site** | **MYB-like sequence** | **MYC** | **Total** |
| --- | --- | --- | --- | --- | --- | --- | --- | --- | --- | --- | --- | --- | --- | --- |
| A | SsCBSD-PB1-3A | 0 | 0 | 6 | 5 | 0 | 0 | 0 | 4 | 1 | 3 | 0 | 3 | 37 |
| A | SsCBSD-PB1-3C-1 | 0 | 0 | 2 | 0 | 1 | 0 | 0 | 5 | 0 | 3 | 0 | 1 | 45 |
| A | SsCBSD-PB1-3C-2 | 1 | 1 | 1 | 1 | 6 | 1 | 0 | 5 | 0 | 1 | 0 | 7 | 29 |
| A | SsCBSD-PB1-3C-4 | 0 | 0 | 4 | 6 | 0 | 1 | 0 | 4 | 1 | 3 | 0 | 4 | 42 |
| A | SsCBSD-PB1-3D-1 | 0 | 2 | 2 | 1 | 0 | 2 | 0 | 7 | 1 | 1 | 0 | 5 | 29 |
| A | SsCBSD-PB1-5A | 0 | 0 | 0 | 0 | 1 | 2 | 0 | 10 | 0 | 4 | 1 | 0 | 45 |
| A | SsCBSD-PB1-5C | 0 | 0 | 4 | 0 | 1 | 1 | 0 | 4 | 1 | 2 | 0 | 3 | 34 |
| A | SsCBSD-PB1-6B | 2 | 0 | 2 | 0 | 0 | 1 | 0 | 2 | 0 | 0 | 0 | 1 | 27 |
| A | SsCBSD-PB1-6D | 1 | 0 | 2 | 0 | 0 | 0 | 1 | 1 | 0 | 0 | 0 | 3 | 25 |
| A | SsCBSD-PB1-7A-1 | 0 | 0 | 0 | 0 | 1 | 2 | 0 | 4 | 0 | 0 | 0 | 3 | 20 |
| A | SsCBSD-PB1-7A-2 | 0 | 0 | 1 | 0 | 1 | 0 | 0 | 3 | 0 | 1 | 0 | 5 | 25 |
| A | SsCBSD-PB1-7D-1 | 0 | 0 | 3 | 0 | 1 | 1 | 0 | 5 | 1 | 1 | 1 | 3 | 30 |
| A | SsCBSD-PB1-7D-2 | 2 | 0 | 1 | 1 | 0 | 0 | 0 | 5 | 0 | 3 | 1 | 2 | 29 |
| B | SsCBS-1C-2 | 3 | 1 | 1 | 0 | 2 | 0 | 0 | 6 | 0 | 3 | 0 | 4 | 30 |
| B | SsCBS-1D-2 | 0 | 0 | 4 | 0 | 0 | 2 | 0 | 5 | 0 | 0 | 2 | 3 | 25 |
| B | SsCBS-2C | 1 | 1 | 2 | 2 | 1 | 0 | 0 | 1 | 0 | 0 | 0 | 1 | 21 |
| B | SsCBS-2D | 2 | 0 | 2 | 0 | 0 | 0 | 0 | 4 | 0 | 0 | 3 | 4 | 18 |
| B | SsCBS-3B-1 | 1 | 0 | 0 | 2 | 1 | 4 | 0 | 7 | 0 | 0 | 1 | 6 | 39 |
| B | SsCBS-3C | 0 | 0 | 2 | 1 | 1 | 3 | 0 | 6 | 0 | 1 | 1 | 5 | 33 |
| B | SsCBS-3D-1 | 0 | 0 | 2 | 2 | 1 | 4 | 0 | 8 | 0 | 1 | 1 | 5 | 40 |
| B | SsCBS-5A | 0 | 0 | 0 | 0 | 0 | 0 | 0 | 0 | 0 | 0 | 0 | 0 | 1 |
| B | SsCBS-5B | 0 | 0 | 0 | 0 | 0 | 0 | 0 | 0 | 0 | 0 | 0 | 0 | 0 |
| B | SsCBS-5C | 0 | 0 | 0 | 0 | 0 | 0 | 0 | 0 | 0 | 0 | 0 | 0 | 2 |
| B | SsCBS-5D | 1 | 1 | 8 | 1 | 1 | 1 | 0 | 1 | 0 | 0 | 0 | 9 | 39 |
| B | SsCBS-CLC-3A | 0 | 0 | 6 | 0 | 0 | 0 | 0 | 1 | 2 | 0 | 0 | 0 | 34 |
| C1 | SsCBS-CLC-4A-1 | 0 | 1 | 2 | 0 | 0 | 2 | 2 | 4 | 2 | 0 | 1 | 2 | 33 |
| C1 | SsCBS-CLC-4B-1 | 0 | 1 | 3 | 0 | 1 | 1 | 0 | 3 | 2 | 1 | 0 | 4 | 45 |
| C1 | SsCBS-CLC-6A-1 | 0 | 0 | 3 | 1 | 0 | 2 | 0 | 6 | 1 | 3 | 0 | 1 | 44 |
| C1 | SsCBS-CLC-6A-2 | 0 | 0 | 3 | 0 | 0 | 3 | 1 | 6 | 1 | 0 | 0 | 5 | 29 |
| C1 | SsCBS-CLC-6B | 0 | 0 | 2 | 1 | 1 | 0 | 0 | 3 | 0 | 1 | 0 | 2 | 27 |
| C1 | SsCBS-CLC-6C | 0 | 0 | 1 | 0 | 0 | 2 | 0 | 5 | 1 | 1 | 2 | 11 | 37 |
| C1 | SsCBS-CLC-6D | 0 | 0 | 4 | 1 | 1 | 0 | 0 | 3 | 0 | 2 | 0 | 1 | 30 |
| C1 | SsCBS-CLC-1A | 0 | 0 | 1 | 1 | 1 | 0 | 1 | 4 | 1 | 0 | 2 | 9 | 34 |
| C2 | SsCBS-CLC-1B | 0 | 1 | 2 | 1 | 1 | 0 | 1 | 4 | 2 | 0 | 2 | 7 | 34 |
| C2 | SsCBS-CLC-3D | 0 | 0 | 2 | 1 | 1 | 1 | 0 | 3 | 1 | 0 | 0 | 2 | 20 |
| C2 | SsCBS-CLC-4A-3 | 1 | 0 | 3 | 1 | 0 | 0 | 0 | 3 | 0 | 1 | 1 | 9 | 45 |
| C2 | SsCBS-CLC-4A-2 | 0 | 0 | 10 | 5 | 0 | 0 | 0 | 2 | 3 | 0 | 0 | 3 | 33 |
| C2 | SsCBS-CLC-4B-2 | 0 | 0 | 1 | 0 | 1 | 0 | 0 | 6 | 0 | 1 | 1 | 2 | 23 |
| C2 | SsCBS-CLC-4C | 1 | 0 | 4 | 1 | 0 | 0 | 0 | 6 | 0 | 1 | 3 | 9 | 53 |
| C2 | SsCBS-CLC-4D | 0 | 0 | 3 | 1 | 1 | 0 | 1 | 2 | 0 | 0 | 0 | 4 | 34 |
| C2 | SsCBS-CLC-5A | 1 | 0 | 4 | 1 | 1 | 2 | 0 | 6 | 0 | 2 | 1 | 5 | 41 |
| C2 | SsCBS-CLC-5B-1 | 0 | 0 | 0 | 0 | 1 | 1 | 0 | 3 | 1 | 0 | 0 | 6 | 27 |
| C2 | SsCBS-CLC-5B-2 | 1 | 0 | 3 | 1 | 0 | 3 | 0 | 6 | 0 | 1 | 1 | 4 | 39 |
| C2 | SsCBS-CLC-5C-1 | 0 | 0 | 0 | 0 | 1 | 1 | 0 | 3 | 1 | 1 | 0 | 5 | 28 |
| C2 | SsCBS-CLC-5C-2 | 1 | 0 | 0 | 1 | 0 | 2 | 0 | 5 | 0 | 1 | 1 | 6 | 41 |
| C2 | SsCBS-CLC-5D-1 | 0 | 0 | 0 | 0 | 1 | 1 | 0 | 3 | 1 | 1 | 0 | 5 | 24 |
| C2 | SsCBS-CLC-5D-2 | 2 | 0 | 3 | 1 | 1 | 1 | 0 | 4 | 0 | 1 | 1 | 3 | 38 |
| C2 | SsCBS-CLC-8B-1 | 0 | 0 | 6 | 0 | 0 | 1 | 0 | 5 | 0 | 1 | 3 | 6 | 34 |
| C2 | SsCBS-CLC-8B-2 | 0 | 0 | 5 | 1 | 0 | 1 | 0 | 5 | 0 | 1 | 3 | 6 | 35 |
| C2 | SsCBS-CLC-8C | 0 | 0 | 5 | 1 | 0 | 1 | 0 | 3 | 0 | 1 | 1 | 8 | 33 |
| C2 | SsCBS-CLC-8D | 0 | 0 | 8 | 0 | 1 | 2 | 0 | 6 | 1 | 2 | 2 | 8 | 39 |
| C2 | SsCBS-4C | 0 | 0 | 3 | 1 | 4 | 1 | 0 | 7 | 0 | 1 | 0 | 1 | 43 |
| D | SsCBS-4D-1 | 0 | 0 | 9 | 4 | 2 | 1 | 0 | 6 | 0 | 1 | 0 | 0 | 48 |
| D | SsTlyc-1A | 1 | 0 | 5 | 1 | 0 | 3 | 0 | 4 | 0 | 0 | 0 | 1 | 33 |
| D | SsTlyc-1B-1 | 1 | 0 | 4 | 2 | 0 | 3 | 0 | 4 | 0 | 0 | 0 | 2 | 52 |
| D | SsTlyc-1B-2 | 0 | 0 | 2 | 1 | 2 | 2 | 0 | 5 | 0 | 0 | 1 | 0 | 24 |
| D | SsTlyc-1C-1 | 0 | 0 | 2 | 0 | 0 | 0 | 2 | 1 | 1 | 0 | 1 | 3 | 25 |
| D | SsTlyc-1C-2 | 1 | 0 | 3 | 1 | 0 | 0 | 1 | 3 | 0 | 1 | 1 | 7 | 33 |
| D | SsTlyc-1D-1 | 0 | 1 | 9 | 1 | 0 | 1 | 0 | 4 | 0 | 1 | 2 | 4 | 42 |
| D | SsTlyc-1D-2 | 0 | 0 | 3 | 1 | 1 | 1 | 0 | 3 | 1 | 0 | 2 | 1 | 25 |
| D | SsTlyc-7A-1 | 0 | 0 | 4 | 3 | 0 | 0 | 2 | 2 | 0 | 0 | 0 | 5 | 32 |
| D | SsTlyc-7B | 2 | 0 | 4 | 0 | 1 | 0 | 1 | 3 | 0 | 0 | 2 | 2 | 28 |
| D | SsTlyc-7C-1 | 0 | 0 | 3 | 1 | 0 | 0 | 0 | 6 | 1 | 1 | 1 | 4 | 27 |
| D | SsTlyc-7C-2 | 1 | 0 | 3 | 1 | 0 | 1 | 1 | 6 | 0 | 2 | 1 | 4 | 41 |
| D | SsCBS-SIS-4C | 0 | 1 | 3 | 0 | 0 | 0 | 0 | 2 | 1 | 1 | 1 | 2 | 23 |
| E | SsCBS-SIS-4D | 0 | 0 | 2 | 0 | 1 | 2 | 0 | 5 | 3 | 0 | 0 | 1 | 42 |
| E | SsCBS-1A | 1 | 0 | 4 | 1 | 2 | 0 | 1 | 1 | 0 | 0 | 1 | 3 | 23 |
| F | SsCBS-1C-1 | 0 | 1 | 6 | 1 | 0 | 0 | 0 | 2 | 0 | 0 | 1 | 5 | 30 |
| F | SsCBS-1D-1 | 0 | 1 | 5 | 1 | 1 | 0 | 0 | 2 | 0 | 0 | 1 | 5 | 31 |
| F | SsCBS-3A-1 | 0 | 1 | 1 | 2 | 2 | 0 | 0 | 5 | 3 | 1 | 1 | 2 | 32 |
| F | SsCBS-3A-2 | 0 | 0 | 3 | 5 | 4 | 1 | 0 | 4 | 2 | 2 | 0 | 1 | 38 |
| F | SsCBS-3B-2 | 0 | 0 | 4 | 2 | 1 | 0 | 1 | 1 | 0 | 2 | 0 | 4 | 33 |
| F | SsCBS-3D-2 | 0 | 0 | 2 | 6 | 3 | 0 | 0 | 3 | 1 | 0 | 1 | 1 | 35 |
| F | SsCBS-4A-1 | 0 | 2 | 2 | 0 | 0 | 0 | 0 | 0 | 1 | 0 | 0 | 4 | 37 |
| F | SsCBS-4A-2 | 1 | 1 | 5 | 2 | 2 | 2 | 0 | 7 | 0 | 0 | 0 | 10 | 56 |
| F | SsCBS-4B | 0 | 2 | 2 | 0 | 0 | 0 | 0 | 0 | 1 | 0 | 0 | 4 | 36 |
| F | SsCBS-4D-2 | 0 | 1 | 3 | 0 | 0 | 0 | 0 | 0 | 1 | 0 | 0 | 4 | 40 |
| F | SsCBS-4D-3 | 1 | 2 | 5 | 1 | 2 | 2 | 0 | 7 | 0 | 0 | 0 | 7 | 51 |
| F | SsCBSD-AMPK1-1A | 0 | 1 | 7 | 4 | 1 | 1 | 0 | 1 | 0 | 0 | 0 | 1 | 31 |
| G | SsCBSD-AMPK1-1D-1 | 0 | 1 | 7 | 4 | 0 | 1 | 0 | 1 | 0 | 0 | 0 | 2 | 29 |
| G | SsCBSD-AMPK1-1D-2 | 0 | 1 | 8 | 1 | 0 | 1 | 0 | 3 | 0 | 1 | 0 | 1 | 34 |
| G | SsCBSD-AMPK1-5D | 1 | 1 | 4 | 2 | 1 | 0 | 0 | 6 | 0 | 3 | 0 | 3 | 30 |
| G | SsCBSDCBS-3A-1 | 0 | 0 | 0 | 0 | 2 | 1 | 0 | 6 | 0 | 0 | 1 | 2 | 23 |
| H | SsCBSDCBS-3A-2 | 1 | 1 | 2 | 0 | 0 | 1 | 1 | 10 | 0 | 2 | 4 | 6 | 41 |
| H | SsCBSDCBS-3A-3 | 2 | 0 | 6 | 0 | 0 | 0 | 1 | 3 | 1 | 0 | 0 | 5 | 26 |
| H | SsCBSDCBS-3B-1 | 0 | 0 | 0 | 0 | 1 | 1 | 0 | 5 | 0 | 0 | 0 | 1 | 22 |
| H | SsCBSDCBS-3B-2 | 0 | 0 | 2 | 0 | 1 | 1 | 0 | 8 | 0 | 2 | 1 | 2 | 32 |
| H | SsCBSDCBS-3B-3 | 0 | 0 | 2 | 0 | 0 | 5 | 0 | 13 | 0 | 1 | 5 | 3 | 40 |
| H | SsCBSDCBS-3C | 0 | 0 | 2 | 0 | 0 | 4 | 0 | 12 | 0 | 1 | 5 | 3 | 31 |
| H | SsCBSDCBS-3D | 0 | 0 | 4 | 0 | 0 | 0 | 0 | 1 | 0 | 0 | 0 | 5 | 21 |
| H | SsCBSDCBS-5A | 0 | 1 | 11 | 0 | 0 | 0 | 0 | 1 | 1 | 0 | 1 | 5 | 43 |
| H | SsCBSDCBS-5B | 0 | 1 | 4 | 0 | 2 | 0 | 1 | 5 | 1 | 2 | 2 | 2 | 35 |
| H | SsCBSDCBS-5C | 0 | 1 | 4 | 0 | 0 | 0 | 1 | 3 | 1 | 2 | 1 | 3 | 35 |
| H | SsCBSDCBS-5D-1 | 0 | 0 | 11 | 3 | 1 | 1 | 0 | 4 | 1 | 2 | 0 | 1 | 42 |
| H | SsCBSDCBS-5D-2 | 0 | 3 | 2 | 0 | 0 | 0 | 0 | 6 | 1 | 5 | 1 | 3 | 42 |

**Table S5** The non-synonymous (Ka) and synonymous (Ks) substitution ratios of *SsCDCP* genes.

| **Gene 1** | **Gene 2** | **Ka** | **Ks** | **Ka/Ks** | **T (MYa)** | **Divergence time** | **Type of Selection** | **Type of Duplication** |
| --- | --- | --- | --- | --- | --- | --- | --- | --- |
| SsCBSD-AMPK1-1A | SsCBSD-AMPK1-1D-2 | 0.005048399 | 0.032516607 | 0.155256032 | 2.665295656 | 2.67 | Negative | Segmental |
| SsCBSD-AMPK1-1A | SsCBSD-AMPK1-1D-1 | 0.004457003 | 0.022761349 | 0.195814547 | 1.865684344 | 1.87 | Negative | Segmental |
| SsCBSD-AMPK1-1D-1 | SsCBSD-AMPK1-1D-2 | 0.00606217 | 0.049319326 | 0.122916723 | 4.042567705 | 4.04 | Negative | Segmental |
| SsCBSDCBS-3A-2 | SsCBSDCBS-3B-3 | 0.002024805 | 0.012075733 | 0.167675548 | 0.98981418 | 0.99 | Negative | Segmental |
| SsCBSDCBS-3A-1 | SsCBSDCBS-3B-1 | 0.00523608 | 0.013789529 | 0.379714207 | 1.130289262 | 1.13 | Negative | Segmental |
| SsCBSDCBS-3A-1 | SsCBSDCBS-3B-2 | 0.010985184 | 0.028815069 | 0.38123052 | 2.361890902 | 2.36 | Negative | Segmental |
| SsCBSD-PB1-3A | SsCBSD-PB1-3C-4 | 0.059028597 | 0.063654254 | 0.927331527 | 5.217561803 | 5.22 | Negative | Segmental |
| SsCBSDCBS-3A-2 | SsCBSDCBS-3C | 0.003935726 | 0.020542195 | 0.191592267 | 1.683786475 | 1.68 | Negative | Segmental |
| SsCBSDCBS-3A-3 | SsCBSDCBS-3D | 0.031983527 | 0.058306375 | 0.548542537 | 4.779211066 | 4.78 | Negative | Segmental |
| SsCBS-3B-1 | SsCBS-3C | 0.015939879 | 0.030892398 | 0.515980631 | 2.53216377 | 2.53 | Negative | Segmental |
| SsCBSDCBS-3B-3 | SsCBSDCBS-3C | 0.003848629 | 0.011928681 | 0.322636626 | 0.977760738 | 0.98 | Negative | Segmental |
| SsCBS-3B-1 | SsCBS-3D-1 | 0.009040793 | 0.031200339 | 0.28976584 | 2.557404836 | 2.56 | Negative | Segmental |
| SsCBS-3B-2 | SsCBS-3D-2 | 0.025510883 | 0.055162208 | 0.462470299 | 4.521492459 | 4.52 | Negative | Segmental |
| SsCBS-3C | SsCBS-3D-1 | 0.005808354 | 0 | 0 | 0 | 0.00 | Negative | Segmental |
| SsCBS-4A-1 | SsCBS-4B | 0.009607818 | 0.042575402 | 0.225665925 | 3.489787049 | 3.49 | Negative | Segmental |
| SsCBS-4A-1 | SsCBS-4D-2 | 0.010639419 | 0.023876674 | 0.445598884 | 1.957104426 | 1.96 | Negative | Segmental |
| SsCBS-4B | SsCBS-4D-2 | 0.012839115 | 0.020980389 | 0.611957921 | 1.719704016 | 1.72 | Negative | Segmental |
| SsCBS-4C | SsCBS-4D-1 | 0 | 0.059216934 | 0 | 4.853847049 | 4.85 | Negative | Segmental |
| SsCBSDCBS-5A | SsCBSDCBS-5B | 0.001036986 | 0.003363234 | 0.308329985 | 0.275674918 | 0.28 | Negative | Segmental |
| SsCBS-5A | SsCBS-5B | 0.028593456 | 0.03002277 | 0.952392339 | 2.460882787 | 2.46 | Negative | Segmental |
| SsCBSDCBS-5A | SsCBSDCBS-5C | 0.002460531 | 0.004146521 | 0.593396601 | 0.33987877 | 0.34 | Negative | Segmental |
| SsCBSDCBS-5A | SsCBSDCBS-5D-2 | 0 | 0 | 0 | 0 | 0.00 | Negative | Segmental |
| SsCBS-5A | SsCBS-5D | 0.00466202 | 0.034409704 | 0.135485607 | 2.820467541 | 2.82 | Negative | Segmental |
| SsCBSD-PB1-5A | SsCBSD-PB1-7A-1 | 0.170452568 | 0.933705182 | 0.18255502 | 76.53321164 | 76.53 | Negative | Segmental |
| SsCBSD-PB1-5A | SsCBSD-PB1-7D-1 | 0.088921081 | 0.636156067 | 0.139778721 | 52.14393992 | 52.14 | Negative | Segmental |
| SsCBSDCBS-5B | SsCBSDCBS-5C | 0.001230265 | 0 | 0 | 0 | 0.00 | Negative | Segmental |
| SsCBS-5B | SsCBS-5D | 0.027756236 | 0.043694102 | 0.635239892 | 3.58148377 | 3.58 | Negative | Segmental |
| SsCBSD-PB1-5C | SsCBSD-PB1-7A-1 | 0.173816347 | 0.846876587 | 0.20524401 | 69.41611369 | 69.42 | Negative | Segmental |
| SsCBSD-PB1-5C | SsCBSD-PB1-7D-1 | 0.086384552 | 0.569410451 | 0.151708758 | 46.67298779 | 46.67 | Negative | Segmental |
| SsCBSD-PB1-6B | SsCBSD-PB1-6D | 0.005488666 | 0.028725387 | 0.191073699 | 2.354539918 | 2.35 | Negative | Segmental |
| SsCBSD-PB1-7A-1 | SsCBSD-PB1-7D-1 | 0 | 0.064521225 | 0 | 5.29 | 5.29 | Negative | Segmental |

**Table S6** The transcript expression (log_2_FC) of 95 *ShCDCPs* in two sugarcane cultivars ROC22 and MT11-610 based on RNA-seq data.

| **group** | **Gene ID** | **Gene name** | **ROC22 (resistant to red stripe)** ^a^ | | |  | **MT11-610 (susceptible to red stripe)** ^a^ | | |
| --- | --- | --- | --- | --- | --- | --- | --- | --- | --- |
|  |  |  | **R24** | **R48** | **R72** |  | **S24** | **S48** | **S72** |
| A | Sspon.03G0000340-1A | ShCBSD-PB1-3A | -0.12 | 0.06 | 0.04 |  | -0.80 | -0.11 | -0.25 |
| A | Sspon.03G0038630-1P | ShCBSD-PB1-3C-1 | 0.30 | 0.53 | 0.10 |  | 0.26 | -0.28 | 0.14 |
| A | Sspon.03G0038630-1C | ShCBSD-PB1-3C-2 | 0.58 | 0.00 | 0.20 |  | 0.00 | 1.22 | 1.22 |
| A | Sspon.03G0000340-2C | ShCBSD-PB1-3C-4 | 0.98 | 0.44 | 1.94 |  | -2.23 | 1.92 | 1.97 |
| A | Sspon.03G0038630-2D | ShCBSD-PB1-3D-1 | 2.32 | 3.08 | 3.03 |  | -2.00 | -2.00 | -0.68 |
| A | Sspon.05G0018330-1A | ShCBSD-PB1-5A | -2.08 | -2.13 | -1.99 |  | -2.28 | -0.72 | -1.07 |
| A | Sspon.05G0018330-2C | ShCBSD-PB1-5C | -0.75 | -0.93 | -0.05 |  | -0.92 | 0.06 | -0.32 |
| A | Sspon.06G0025100-1B | ShCBSD-PB1-6B | NA | NA | NA |  | NA | NA | NA |
| A | Sspon.06G0025100-3D | ShCBSD-PB1-6D | NA | NA | NA |  | NA | NA | NA |
| A | Sspon.05G0018330-1P | ShCBSD-PB1-7A-1 | 1.12 | 1.11 | 1.03 |  | 1.53 | 2.21 | 1.47 |
| A | Sspon.07G0019280-1A | ShCBSD-PB1-7A-2 | NA | NA | NA |  | NA | NA | NA |
| A | Sspon.05G0018450-2D | ShCBSD-PB1-7D-1 | 1.12 | 1.11 | 1.03 |  | 1.53 | 2.21 | 1.47 |
| A | Sspon.07G0019280-2D | ShCBSD-PB1-7D-2 | 0.29 | 0.30 | 0.11 |  | -0.08 | 0.89 | 0.09 |
| B | Sspon.01G0057070-1C | ShCBS-1C-2 | 0.50 | 0.35 | -0.13 |  | -1.28 | -2.14 | -2.56 |
| B | Sspon.01G0057070-2D | ShCBS-1D-2 | -0.17 | -0.11 | -0.03 |  | -0.81 | -1.65 | -1.81 |
| B | Sspon.02G0049550-1C | ShCBS-2C | 0.46 | 0.22 | -0.34 |  | 0.08 | -0.64 | -1.03 |
| B | Sspon.02G0049550-2D | ShCBS-2D | -0.97 | -1.57 | -1.94 |  | -0.54 | -0.55 | -1.81 |
| B | Sspon.03G0032200-1B | ShCBS-3B-1 | 0.26 | 0.00 | 0.00 |  | 0.00 | 0.14 | 0.00 |
| B | Sspon.03G0032200-2C | ShCBS-3C | NA | NA | NA |  | NA | NA | NA |
| B | Sspon.03G0032200-3D | ShCBS-3D-1 | NA | NA | NA |  | NA | NA | NA |
| B | Sspon.05G0014080-1A | ShCBS-5A | -0.52 | -1.82 | -0.75 |  | -1.05 | -1.07 | -1.24 |
| B | Sspon.05G0014080-2B | ShCBS-5B | -0.49 | -1.40 | -0.53 |  | -0.56 | -1.69 | -0.65 |
| B | Sspon.05G0014080-3C | ShCBS-5C | -1.01 | -3.32 | -2.50 |  | -2.12 | -2.03 | -1.87 |
| B | Sspon.05G0014080-4D | ShCBS-5D | -0.68 | -1.06 | -0.94 |  | -0.71 | -0.62 | -0.77 |
| C1 | Sspon.03G0004960-1A | ShCBS-CLC-3A | 0.28 | -0.46 | -0.39 |  | -0.44 | -0.74 | -0.82 |
| C1 | Sspon.04G0004760-1A | ShCBS-CLC-4A-1 | -1.20 | -1.66 | -1.29 |  | -1.61 | -1.24 | -0.70 |
| C1 | Sspon.04G0004760-2B | ShCBS-CLC-4B-1 | -0.82 | -1.20 | -0.56 |  | -1.45 | -1.28 | -0.53 |
| C1 | Sspon.06G0000980-1A | ShCBS-CLC-6A-1 | 1.46 | 1.23 | 0.92 |  | -0.70 | -0.69 | -0.74 |
| C1 | Sspon.06G0000980-1P | ShCBS-CLC-6A-2 | -0.49 | -0.73 | -0.86 |  | -0.76 | -0.64 | -0.55 |
| C1 | Sspon.06G0000980-2B | ShCBS-CLC-6B | 0.30 | 0.21 | 0.07 |  | -0.64 | -0.48 | -0.52 |
| C1 | Sspon.06G0000980-3C | ShCBS-CLC-6C | 0.61 | 0.81 | 0.44 |  | -0.51 | -0.57 | -0.38 |
| C1 | Sspon.06G0000980-4D | ShCBS-CLC-6D | -0.05 | -1.17 | 0.25 |  | 1.15 | -0.72 | -2.20 |
| C2 | Sspon.01G0025410-1A | ShCBS-CLC-1A | 0.13 | 0.19 | 0.36 |  | -0.25 | 0.01 | -0.01 |
| C2 | Sspon.01G0025410-2B | ShCBS-CLC-1B | 0.02 | 0.17 | 0.40 |  | -0.14 | -0.14 | -0.30 |
| C2 | Sspon.03G0029010-3D | ShCBS-CLC-3D | -0.66 | 0.00 | -2.36 |  | 0.00 | 0.00 | 0.00 |
| C2 | Sspon.04G0009220-1A | ShCBS-CLC-4A-3 | 0.02 | 0.63 | 1.03 |  | 0.43 | 1.18 | 1.12 |
| C2 | Sspon.04G0016160-1A | ShCBS-CLC-4A-2 | 0.52 | -0.23 | 0.69 |  | -1.23 | -1.57 | -1.05 |
| C2 | Sspon.04G0016160-2B | ShCBS-CLC-4B-2 | 1.26 | 0.65 | 1.42 |  | -1.13 | -1.57 | -1.25 |
| C2 | Sspon.04G0009220-3C | ShCBS-CLC-4C | 0.14 | 0.26 | 0.70 |  | 0.47 | 0.83 | 0.98 |
| C2 | Sspon.04G0016160-3D | ShCBS-CLC-4D | -0.09 | -1.00 | 0.00 |  | -0.99 | -1.81 | -1.72 |
| C2 | Sspon.04G0009220-1P | ShCBS-CLC-5A | 0.55 | 0.09 | -0.14 |  | 0.38 | 1.55 | 1.86 |
| C2 | Sspon.05G0022270-1B | ShCBS-CLC-5B-1 | -0.10 | -0.51 | -0.18 |  | -0.36 | -1.37 | -1.13 |
| C2 | Sspon.04G0009220-2B | ShCBS-CLC-5B-2 | 0.00 | 0.00 | 0.00 |  | -1.71 | -0.27 | 0.12 |
| C2 | Sspon.05G0022270-2C | ShCBS-CLC-5C-1 | 0.16 | 0.27 | -0.05 |  | -0.47 | -1.68 | -0.75 |
| C2 | Sspon.04G0009220-2P | ShCBS-CLC-5C-2 | 1.07 | 1.07 | 0.67 |  | 0.36 | 1.23 | 1.62 |
| C2 | Sspon.05G0022270-3D | ShCBS-CLC-5D-1 | -0.17 | -0.33 | 0.08 |  | -1.04 | -1.51 | -1.38 |
| C2 | Sspon.04G0009220-4D | ShCBS-CLC-5D-2 | 1.24 | 1.11 | 0.96 |  | 0.63 | 1.60 | 1.75 |
| C2 | Sspon.08G0019260-1T | ShCBS-CLC-8B-1 | 1.44 | 0.98 | 1.09 |  | 0.73 | 1.13 | 0.80 |
| C2 | Sspon.08G0019260-1B | ShCBS-CLC-8B-2 | NA | NA | NA |  | NA | NA | NA |
| C2 | Sspon.08G0019260-2C | ShCBS-CLC-8C | 0.75 | 0.25 | 0.69 |  | -1.07 | 0.58 | -0.62 |
| C2 | Sspon.08G0019260-3D | ShCBS-CLC-8D | 0.87 | 0.66 | 0.39 |  | 0.33 | 0.95 | 0.42 |
| D | Sspon.04G0030670-1C | ShCBS-4C | 0.48 | 0.09 | -0.05 |  | -0.21 | -0.27 | -0.65 |
| D | Sspon.04G0030670-1P | ShCBS-4D-1 | 2.04 | 2.05 | 0.41 |  | -0.99 | -2.08 | -1.05 |
| D | Sspon.01G0001530-1A | ShTlyc-1A | 0.36 | 0.33 | 0.49 |  | -0.03 | -0.04 | -0.53 |
| D | Sspon.01G0046430-1B | ShTlyc-1B-1 | 0.40 | -0.39 | -0.42 |  | -0.66 | 0.45 | 0.78 |
| D | Sspon.01G0001530-2B | ShTlyc-1B-2 | 0.38 | 0.36 | 1.11 |  | -0.12 | -0.36 | -0.31 |
| D | Sspon.01G0044900-2C | ShTlyc-1C-1 | 0.89 | 0.86 | 0.74 |  | -0.17 | -0.04 | -0.50 |
| D | Sspon.07G0010520-1P | ShTlyc-1C-2 | 0.61 | 0.45 | 0.84 |  | 0.41 | 0.24 | 0.21 |
| D | Sspon.01G0044900-1P | ShTlyc-1D-1 | 1.20 | 1.15 | 0.78 |  | -0.10 | -0.38 | -0.52 |
| D | Sspon.01G0001530-1P | ShTlyc-1D-2 | -0.01 | -0.23 | 0.08 |  | -0.06 | -0.30 | -0.46 |
| D | Sspon.07G0010520-1A | ShTlyc-7A-1 | 0.29 | 0.46 | 0.59 |  | 0.21 | 0.25 | 0.36 |
| D | Sspon.07G0010520-2B | ShTlyc-7B | NA | NA | NA |  | NA | NA | NA |
| D | Sspon.07G0010520-3C | ShTlyc-7C-1 | 1.19 | 0.56 | 0.91 |  | 0.09 | 0.04 | -0.20 |
| D | Sspon.07G0031220-1C | ShTlyc-7C-2 | -3.30 | 0.25 | 0.67 |  | 0.99 | -0.53 | 0.96 |
| E | Sspon.04G0034040-1C | ShCBS-SIS-4C | 0.22 | 0.86 | 0.37 |  | -0.51 | -1.62 | -0.42 |
| E | Sspon.04G0013500-2D | ShCBS-SIS-4D | -0.31 | -1.20 | -0.25 |  | -1.41 | -1.41 | -0.95 |
| F | Sspon.01G0020640-1A | ShCBS-1A | 0.10 | 0.54 | -0.31 |  | 1.20 | 1.26 | 0.25 |
| F | Sspon.01G0020640-2C | ShCBS-1C-1 | -0.38 | 0.03 | -0.95 |  | 1.77 | 1.24 | 0.98 |
| F | Sspon.01G0020640-3D | ShCBS-1D-1 | -0.18 | 0.18 | -0.65 |  | 1.81 | 1.83 | 0.98 |
| F | Sspon.03G0000490-1A | ShCBS-3A-1 | 0.70 | -0.65 | 1.34 |  | 1.52 | 1.03 | 1.52 |
| F | Sspon.03G0000490-1P | ShCBS-3A-2 | 1.80 | 0.63 | 2.02 |  | 0.00 | 1.16 | -0.21 |
| F | Sspon.03G0000490-2B | ShCBS-3B-2 | 1.10 | 0.00 | 2.58 |  | 1.00 | 0.00 | 1.72 |
| F | Sspon.03G0000490-3D | ShCBS-3D-2 | 0.15 | -0.43 | 0.65 |  | 0.21 | 0.46 | 0.57 |
| F | Sspon.04G0017870-1A | ShCBS-4A-1 | -1.00 | -3.32 | -1.32 |  | 2.25 | 2.64 | 5.86 |
| F | Sspon.04G0007500-1A | ShCBS-4A-2 | 0.00 | 0.00 | 2.38 |  | 0.00 | 0.00 | 0.00 |
| F | Sspon.04G0017870-2B | ShCBS-4B | -3.08 | -3.30 | 0.00 |  | 2.51 | 2.58 | 5.76 |
| F | Sspon.04G0017870-3D | ShCBS-4D-2 | -1.30 | -2.65 | -2.79 |  | 0.00 | 0.00 | 0.00 |
| F | Sspon.04G0007500-2D | ShCBS-4D-3 | NA | NA | NA |  | NA | NA | NA |
| G | Sspon.01G0033510-1A | ShCBSD-AMPK1-1A | -0.22 | -0.34 | -0.20 |  | -0.54 | -0.55 | -0.50 |
| G | Sspon.01G0033510-1P | ShCBSD-AMPK1-1D-1 | -0.12 | -0.21 | -0.17 |  | -0.24 | -0.31 | -0.23 |
| G | Sspon.01G0033510-2D | ShCBSD-AMPK1-1D-2 | 0.27 | 0.30 | 0.35 |  | 0.01 | -0.21 | -0.07 |
| G | Sspon.05G0012300-2D | ShCBSD-AMPK1-5D | 0.02 | -0.21 | -0.22 |  | -0.56 | -0.64 | -1.05 |
| H | Sspon.03G0010600-1A | ShCBSDCBS-3A-1 | -0.57 | -1.47 | 0.32 |  | 0.06 | -0.09 | 1.51 |
| H | Sspon.03G0012400-1A | ShCBSDCBS-3A-2 | 0.09 | 0.01 | -0.23 |  | -0.76 | -1.07 | -0.58 |
| H | Sspon.03G0000380-1A | ShCBSDCBS-3A-3 | 0.00 | 0.00 | -0.42 |  | 0.00 | 0.00 | -0.42 |
| H | Sspon.03G0010600-1P | ShCBSDCBS-3B-1 | -0.84 | -1.97 | 0.27 |  | -0.20 | -2.83 | 1.03 |
| H | Sspon.03G0010600-2B | ShCBSDCBS-3B-2 | -2.05 | -0.57 | 0.53 |  | -0.52 | 0.05 | 1.57 |
| H | Sspon.03G0012400-2B | ShCBSDCBS-3B-3 | -0.56 | -0.58 | -0.86 |  | -0.68 | -1.03 | -0.83 |
| H | Sspon.03G0012400-3C | ShCBSDCBS-3C | -0.31 | -1.17 | -0.96 |  | -0.88 | -1.75 | -0.63 |
| H | Sspon.03G0000380-1P | ShCBSDCBS-3D | 0.00 | 0.00 | 0.00 |  | 0.00 | 1.42 | 2.00 |
| H | Sspon.05G0013010-1A | ShCBSDCBS-5A | 2.05 | 1.63 | 0.90 |  | 0.00 | 0.00 | 0.71 |
| H | Sspon.05G0013010-2B | ShCBSDCBS-5B | NA | NA | NA |  | NA | NA | NA |
| H | Sspon.05G0013010-3C | ShCBSDCBS-5C | NA | NA | NA |  | NA | NA | NA |
| H | Sspon.03G0000380-2D | ShCBSDCBS-5D-1 | NA | NA | NA |  | NA | NA | NA |
| H | Sspon.05G0013010-4D | ShCBSDCBS-5D-2 | NA | NA | NA |  | NA | NA | NA |

^a^ NA, not available.

**Table S7** The transcript expression of nine *ShCDCPs* in two sugarcane cultivars ROC22 and MT11-610 under multiple stressors base on RT-qPCR data

| **Under *Aaa* infection** | | |  | | | | | | | |
| --- | --- | --- | --- | --- | --- | --- | --- | --- | --- | --- |
| **gene name** | **ROC22 (leaf scald-resistant)** | | | | |  | **MT11-610 (susceptible to red stripe)** | | | |
|  | **R0** | **R24** | | **R48** | **R72** |  | **S0** | **S24** | **S48** | **S72** |
| ShCBSD-PB1-3A | 1.002 b | 0.988 b | | 1.310 a | 1.125 ab |  | 1.002 b | 0.921 b | 1.395 a | 1.481 a |
| ShCBSD-PB1-3C-4 | 1.000 c | 0.865 d | | 1.453 a | 1.235 b |  | 1.001 b | 1.053 b | 1.406 a | 1.619 a |
| ShCBSD-PB1-5A | 1.001 a | 0.255 c | | 0.472 b | 0.532 b |  | 1.000 a | 0.925 a | 1.055 a | 1.164 a |
| ShCBSD-PB1-7A-1 | 1.002 a | 0.253 c | | 0.410 b | 0.488 b |  | 1.002 b | 0.789 c | 0.918 b | 1.144 a |
| ShCBS-1D-2 | 1.003 b | 0.804 c | | 1.459 a | 1.406 a |  | 1.002 b | 1.239 a | 1.306 a | 1.516 a |
| ShCBS-4C | 1.007 a | 0.671 b | | 0.682 b | 0.452 c |  | 1.001 a | 0.234 c | 0.565 b | 1.061 a |
| ShCBS-4D-1 | 1.011 d | 4.578 b | | 6.152 a | 4.222 c |  | 1.000 c | 1.152 c | 2.215 b | 2.468 a |
| ShCBS-5D | 1.003 b | 1.186 a | | 1.130 a | 0.785 c |  | 1.000 a | 1.006 a | 1.064 a | 0.890 b |
| ShCBSDCBS-5A | 1.001 c | 1.690 b | | 2.539 a | 1.456 b |  | 1.000 c | 0.939 c | 1.700 a | 1.390 b |
|  | | |  | | | | | | | |
| **Under NaCl stress** | | |  | | | | | | | |
| **gene name** | **ROC22** | | | | |  | **MT11-610** | | | |
|  | **R0** | **R6** | | **R12** | **R24** |  | **S0** | **S6** | **S12** | **S24** |
| ShCBSD-PB1-3A | 1.000 c | 2.874 a | | 1.665 b | 2.432 a |  | 1.000 c | 1.183 b | 1.561 a | 1.254 b |
| ShCBSD-PB1-3C-4 | 1.006 c | 2.193 a | | 1.659 b | 2.454 a |  | 1.001 c | 1.209 b | 1.421 a | 1.269 b |
| ShCBSD-PB1-5A | 1.014 a | 0.693 b | | 0.777 b | 0.487 b |  | 1.000 a | 0.323 b | 0.272 c | 0.170 d |
| ShCBSD-PB1-7A-1 | 1.003 a | 0.726 b | | 0.687 b | 0.502 b |  | 1.001 a | 1.033 a | 0.736 b | 0.493 c |
| ShCBS-1D-2 | 1.001 b | 1.492 a | | 1.159 a | 1.632 a |  | 1.001 a | 0.300 b | 0.240 c | 0.132 d |
| ShCBS-4C | 1.004 c | 3.815 a | | 1.800 b | 1.474 bc |  | 1.001 c | 2.523 a | 2.676 a | 2.083 b |
| ShCBS-4D-1 | 1.001 c | 1.982 a | | 1.717 b | 0.991 c |  | 1.000 b | 1.176 a | 0.612 c | 0.205 d |
| ShCBS-5D | 1.000 d | 4.053 b | | 3.592 c | 8.205 a |  | 1.001 bc | 1.131 b | 0.927 c | 1.304 a |
| ShCBSDCBS-5A | 1.002 c | 2.986 a | | 2.934 a | 2.268 b |  | 1.001 a | 0.848 b | 0.485 c | 0.201 d |
|  | | |  | | | | | | | |
| **Under PEG6000 stress** | | |  | | | | | | | |
| **gene name** | **ROC22** | | | | |  | **MT11-610** | | | |
|  | **R0** | **R3** | | **R6** | **R12** |  | **S0** | **S3** | **S6** | **S12** |
| ShCBSD-PB1-3A | 1.000 c | 1.027 c | | 1.675 b | 2.595 a |  | 1.004 c | 0.715 d | 1.509 b | 2.521 a |
| ShCBSD-PB1-3C-4 | 1.000 c | 1.025 c | | 1.713 b | 2.102 a |  | 1.001 c | 0.711 d | 1.564 b | 2.344 a |
| ShCBSD-PB1-5A | 1.002 a | 0.632 b | | 1.071 a | 0.632 b |  | 1.007 a | 0.476 b | 0.317 b | 0.477 b |
| ShCBSD-PB1-7A-1 | 1.001 a | 0.852 b | | 0.391 d | 0.541 c |  | 1.006 a | 0.480 b | 0.383 b | 0.466 b |
| ShCBS-1D-2 | 1.003 c | 0.468 d | | 1.373 b | 2.486 a |  | 1.005 b | 0.785 c | 0.903 b | 1.706 a |
| ShCBS-4C | 1.000 c | 0.681 d | | 1.529 b | 2.161 a |  | 1.005 d | 2.668 c | 3.990 b | 7.076 a |
| ShCBS-4D-1 | 1.000 c | 1.452 a | | 1.381 a | 1.186 b |  | 1.000 b | 0.833 c | 1.521 a | 1.650 a |
| ShCBS-5D | 1.000 c | 2.196 b | | 2.418 b | 3.420 a |  | 1.001 b | 1.791 a | 0.836 c | 1.218 b |
| ShCBSDCBS-5A | 1.000 c | 1.849 b | | 3.354 a | 3.897 a |  | 1.001 c | 0.501 d | 1.513 a | 1.268 b |
|  | | |  | | | | | | | |
| **Under exogenous SA stress** | | |  | | | | | | | |
| **gene name** | **ROC22** | | | | |  | **MT11-610** | | | |
|  | **R0** | **R6** | | **R12** | **R24** |  | **S0** | **S6** | **S12** | **S24** |
| ShCBSD-PB1-3A | 1.000 a | 0.215 c | | 0.221 c | 0.937 b |  | 1.003 b | 1.004 b | 1.170 a | 1.259 a |
| ShCBSD-PB1-3C-4 | 1.028 d | 1.696 b | | 2.389 a | 1.363 c |  | 1.000 a | 0.667 c | 0.721 c | 0.800 b |
| ShCBSD-PB1-5A | 1.000 a | 0.721 b | | 0.675 b | 0.698 b |  | 1.001 a | 0.333 c | 0.314 c | 0.446 b |
| ShCBSD-PB1-7A-1 | 1.000 a | 0.741 b | | 0.607 b | 0.705 b |  | 1.001 a | 0.288 c | 0.286 c | 0.649 b |
| ShCBS-1D-2 | 1.002 b | 1.828 a | | 0.949 c | 0.527 c |  | 1.000 a | 0.299 b | 0.932 a | 0.995 a |
| ShCBS-4C | 1.002 b | 1.249 b | | 2.343 a | 1.112 b |  | 1.000 a | 0.168 d | 0.324 c | 0.873 b |
| ShCBS-4D-1 | 1.003 c | 6.914 a | | 5.193 b | 1.165 c |  | 1.000 c | 0.306 d | 1.147 b | 2.046 a |
| ShCBS-5D | 1.008 d | 11.307 a | | 3.826 b | 2.292 c |  | 1.001 a | 0.387 b | 0.346 b | 0.392 b |
| ShCBSDCBS-5A | 1.000 d | 4.634 a | | 3.217 b | 1.420 c |  | 1.000 a | 0.245 c | 0.861 b | 0.856 b |


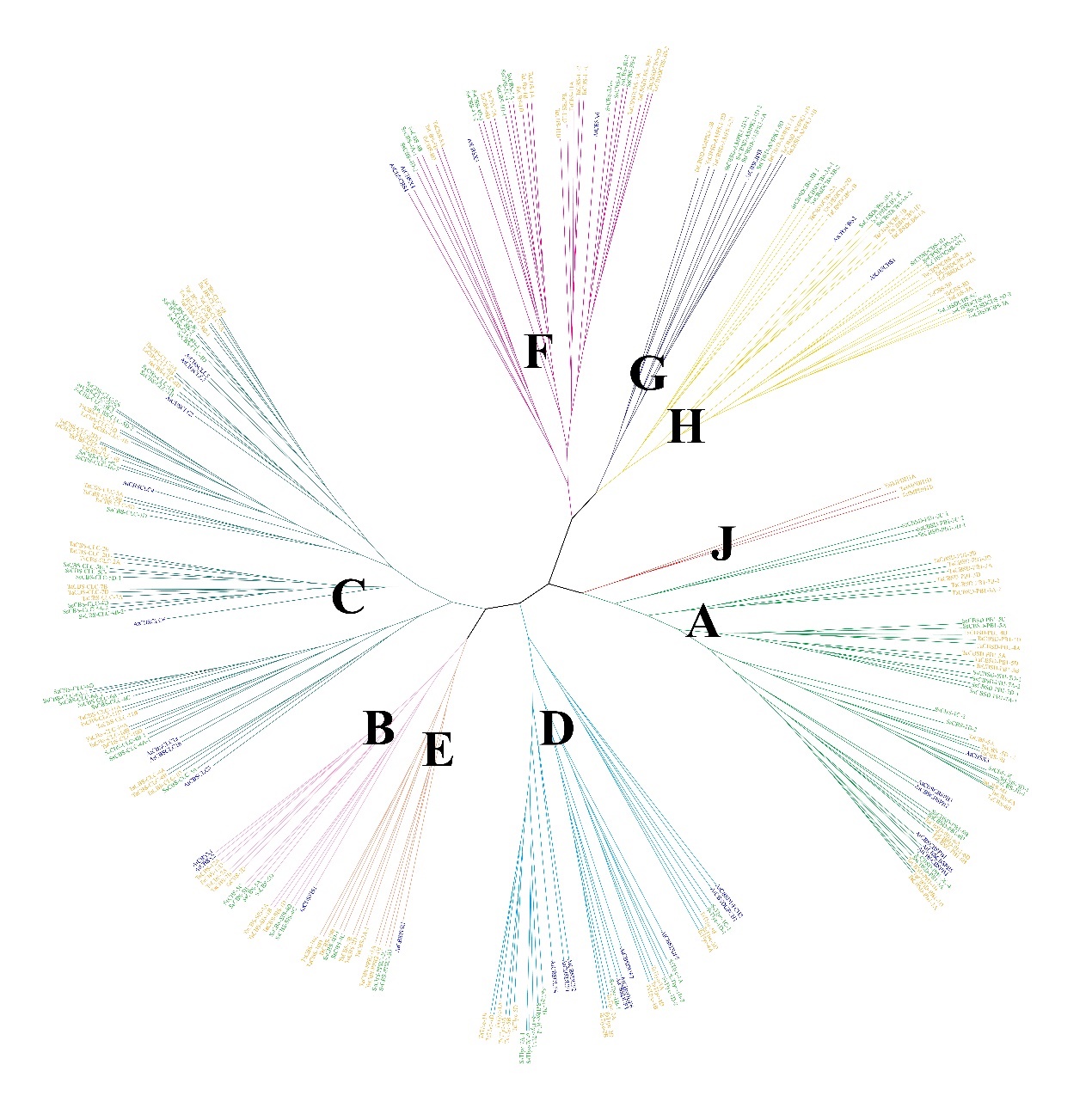


**Figure S1** Unrooted phylogenetic tree was constructed with the maximum likelihood (ML) method based on 256 CDCP family members in *Saccharum spontaneum* (95 *SsCDCPs*), *Arabidopsis thaliana* (34 *AtCDCPs*), and *Triticum aestivum* (127 *TaCDCPs*). Bootstrap analysis of 1000 replicates was conducted.
